# APOB100 transgenic mice exemplify how the systemic circulation content may affect the retina without altering retinal cholesterol input

**DOI:** 10.1007/s00018-023-05056-4

**Published:** 2024-01-22

**Authors:** Nicole El-Darzi, Natalia Mast, Yong Li, Irina A. Pikuleva

**Affiliations:** Department of Ophthalmology and Visual Science, Case Western Reserve University, Cleveland, OH 44106 USA

**Keywords:** Retinal pigment epithelium, Bruch’s membrane, Cholesterol, HDL subpopulation, Age-related macular degeneration

## Abstract

Apolipoprotein B (APOB) is a constituent of unique lipoprotein particles (LPPs) produced in the retinal pigment epithelium (RPE), which separates the neural retina from Bruch’s membrane (BrM) and choroidal circulation. These LPPs accumulate with age in BrM and contribute to the development of age-related macular degeneration, a major blinding disease. The APOB100 transgenic expression in mice, which unlike humans lack the full-length APOB100, leads to lipid deposits in BrM. Herein, we further characterized APOB100 transgenic mice. We imaged mouse retina in vivo and assessed chorioretinal lipid distribution, retinal sterol levels, retinal cholesterol input, and serum content as well as tracked indocyanine green-bound LPPs in mouse plasma and retina after an intraperitoneal injection. Retinal function and differentially expressed proteins were also investigated. APOB100 transgenic mice had increased serum LDL content and an additional higher density HDL subpopulation; their retinal cholesterol levels (initially decreased) became normal with age. The LPP cycling between the RPE and choroidal circulation was increased. Yet, LPP trafficking from the RPE to the neural retina was limited, and total retinal cholesterol input did not change. There were lipid deposits in the RPE and BrM, and retinal function was impaired. Retinal proteomics provided mechanistic insights. Collectively, our data suggested that the serum LDL/HDL ratio may not affect retinal pathways of cholesterol input as serum LPP load is mainly handled by the RPE, which offloads LPP excess to the choroidal circulation rather than neural retina. Different HDL subpopulations should be considered in studies linking serum LPPs and age-related macular degeneration.

## Introduction

**Fig. 1:**
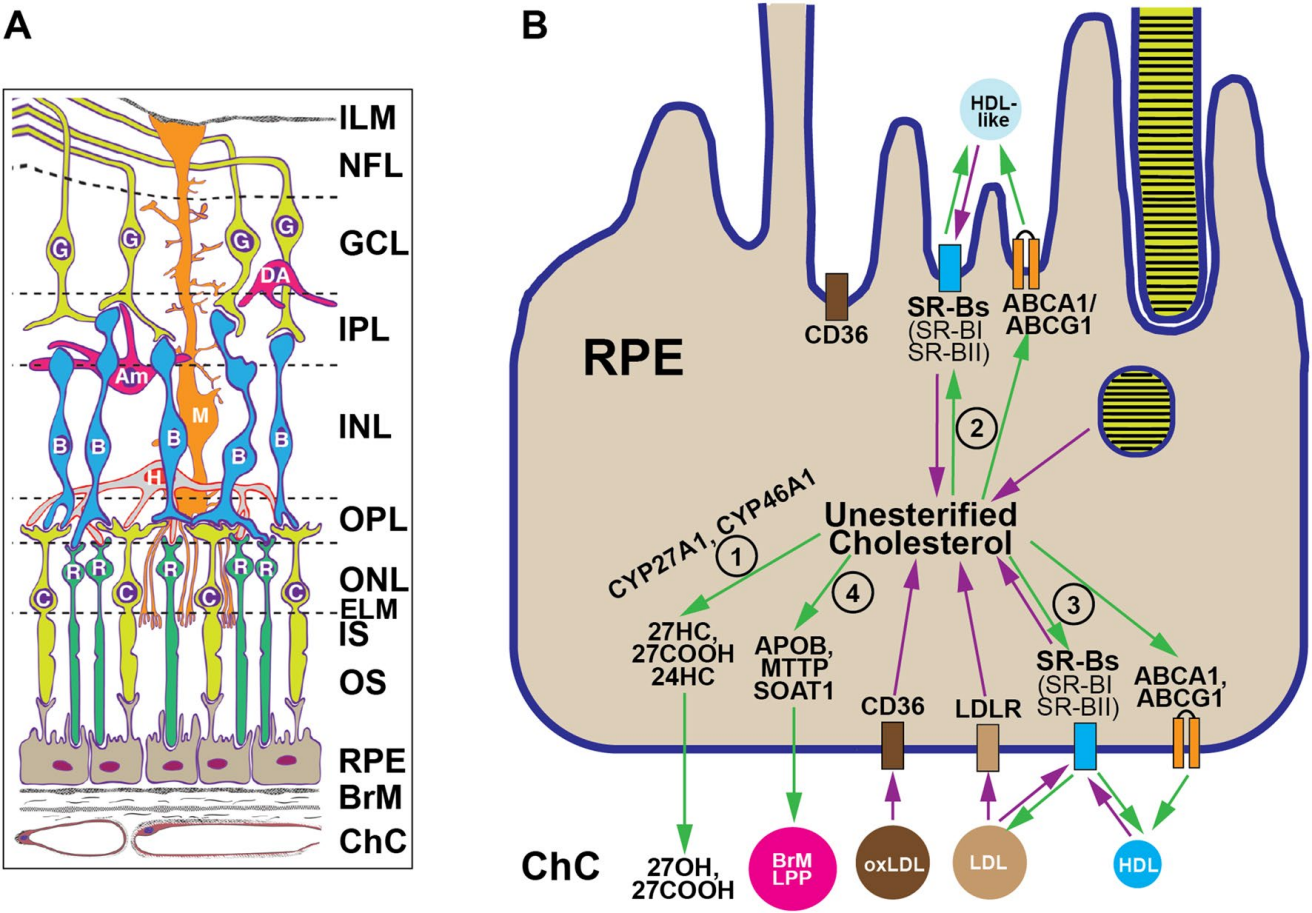
Retina essentials. **A** Schematic representation of retinal structure (taken from [42]). Neural retina: ILM, internal limiting membrane; NFL, nerve fiber layer; G, ganglion cells; GCL, the ganglion cell layer; DA, displaced amacrine cell; IPL, the inner plexiform layer; Am, amacrine cells; M, Muller cell; B, bipolar cells; H, horizontal cell; INL, the inner nuclear layer; OPL, the outer plexiform layer; R, rod photoreceptors; C, cone photoreceptors; ONL, the outer nuclear layer; ELM, external limiting membrane; IS, the photoreceptor inner segments; OS, the photoreceptor outer segments. RPE, retinal pigment epithelium: its tight junctions were omitted for illustrative clarity. BrM, Bruch’s membrane; ChC, choroidal circulation. **B** The RPE pathways of cholesterol input (purple arrows) and output (green arrows and numbered circles). The latter include: (1) oxysterol production; (2) and (3) apical and basal cholesterol efflux, respectively; and (4) elimination on Bruch’s membrane lipoprotein particles (BrM LPP). The oxysterol production in the RPE as well as in the neural retina is mediated by CYP27A1 (cytochrome P450 27A1), which converts cholesterol to 27-hydroxycholesterol (27HC) or 5-cholestenoic acid (27COOH) and by CYP46A1 (cytochrome P450 46A1), which generates 24-hydroxycholesterol (24HC) [8]. Apical and basal RPE efflux of unesterified cholesterol involves the transmembrane ABCA1 and ABCG1 (ATP-binding cassette A1 and G1, respectively) transporters, which transfer cholesterol to extracellular acceptors [5, 42, 117–120]. The expression of both transporters was reported to be higher in the apical than basal RPE membrane [121]. The SR-BI receptors interact with both LDL and HDL and mediate the bi-directional flux of unesterified cholesterol between cells and these LPPs. In addition, SR-BI promotes selective cellular uptake of esterified cholesterol by interacting with HDL [90]. Finally, the RPE expresses both APOB (apolipoprotein B), MTTP (microsomal triglyceride transfer protein), and a cholesterol esterifying enzyme SOAT1 [14]] and secretes esterified cholesterol-rich LPPs into BrM [12–16]. These LPPs (called BrM LPPs) are distinct from the LPPs in the systemic circulation both in protein and lipid composition as besides APOB, they contain APOE, APOA1, APOCI, APOC2, and APOJ and are large like VLDL, yet rich in EC like LDL [12, 13, 15, 16, 18, 122]. oxLDL, oxidized LDL. The basal RPE infoldings [123] were omitted for illustrative clarity

The neural retina is a multi-layered sensory tissue in the back of the eye (Fig. 1A), which receives visual stimuli and transmits them to the brain for subsequent processing. The retinal pigment epithelium (RPE), a monolayer of polarized cuboidal cells, lies beneath the neural retina with its apical side facing the retinal photoreceptor layer and basal side facing the choroidal circulation (ChC). On the apical side, the RPE microvilli have tight contacts with photoreceptor tips, forming a complex, which is often called “the retina,” which also includes all other layers of the neural retina (Fig. 1A). On the basal side, the RPE rests on the basal lamina of an extracellular matrix, which is underlied by a trilaminar Bruch’s membrane (BrM). BrM acts as a planar vessel wall and separates the neural retina-RPE complex from the ChC [1, 2]. ChC accounts for 85% of the blood supply to the retina and provides the RPE and photoreceptors with oxygen, nutrients, vitamins, salts, and lipids, while removing retinal waste products [3].

The RPE tight junctions form the outer retinal-blood barrier and prevent diffusion of large molecules from the choroid to the retina. Therefore, cholesterol from lipoprotein particles (LPPs) in the choroidal (systemic) circulation is delivered to the RPE via different receptors (LDLR, CD36, SR-BI, and SR-BII) (Fig. 1B) present on the RPE basal membrane [4–7]. Cholesterol can also enter the RPE from the apical side, i.e., from the neural retina, via daily photoreceptor phagocytosis and delivery on HDL-like particles that transport lipids between different retinal cells [5]. Once in the RPE, the LPPs, HDL-like particles, and fragments of the photoreceptor outer segments are processed by their corresponding specific mechanisms, and unesterified cholesterol (UC) is ultimately released from the RPE lysosomes. Evidence exist that this cholesterol could then enter four different pathways (Fig. 1B): (1) local metabolism to oxysterols [8, 9]; (2) apical efflux into subretinal space [5]; (3) basal efflux towards the choroid [10, 11]; and (4) esterification with subsequent basolateral release as a constituent of apolipoprotein B (APOB)-containing LPPs, a unique LPPs called BrM LPPs [12–15].

Importantly, cholesterol-rich BrM LPPs accumulate with age in BrM and become components of soft drusen and basal linear deposits [16–18], the pathognomonic lesions for age-related macular degeneration (AMD) [19], a major cause of legal blindness in the elderly [20]. Yet, RPE mechanisms that control cholesterol routing to different pathways and their quantitative significance are currently unknown. This knowledge is necessary to understand how cholesterol becomes a major component of drusen and subretinal drusenoid deposits (another AMD hallmark lesion) [21–23], and thus find preventions or treatments at an earlier AMD stage.

In humans and mice, APOB is encoded by one gene, which is mainly expressed in the liver and intestine and undergoes intestinal mRNA editing in humans and both intestinal and hepatic mRNA editing in mice. As a result, two isoforms (full-length APOB100 and its N-terminal splice variant APOB48) are produced in humans, while mostly APOB48 is produced in mice [24]. APOB100 and APOB48 serve as the major scaffold proteins in LPPs: VLDL, IDL, and LDL contain APOB100, whereas chylomicrons and chylomicron remnants contain APOB48 [25]. The APOB isoform in BrM LPPs was not determined [12]. However, several RPE cell lines were found to synthesize and secrete APOB100 [15, 26]. APOB100 is recognized by the LDL receptor, whereas APOB48 does not have the LDL receptor binding domain; hence the routes of the APOB100- and APOB48-containing LPP clearance from the systemic circulation are different [27].

Previously, APOB100 transgenic mice (APOB100 Tg) were evaluated as an AMD model and shown to have changes in the RPE-BrM region [28–31]. In the present study, we further characterized this genotype and ascertained whether its increased plasma LDL content [32, 33] alters retinal cholesterol input. This input, besides the RPE uptake of cholesterol-containing serum LPPs [6, 34], also includes in situ cholesterol biosynthesis [35], which in mice, accounts for 72–78% of total retinal cholesterol input [36, 37]. We obtained the data that provided novel mechanistic insights into retinal cholesterol maintenance and how the components of the systemic circulation may affect this process.

## Materials and methods

### Animals

Female and male hemizygous APOB100 Tg mice on the C57BL/6N background were from Taconic Biosciences, Inc. (Germantown, NY, USA, #1004). This model mainly expresses human APOB100 in the liver and has very low APOB100 expression in the small intestine and heart [32]. In the plasma of APOB100 Tg mice, human APOB100 is largely present on LDL, and plasma LDL levels are elevated in these animals [32]. In addition, small amounts of APOB100 are also found in APOB100 Tg mice on VLDL and HDL [32]. APOB100 Tg mice had the *Crbl*^*rd8*^ mutation, which was bred out from our colony by crosses with *Crbl*^*rd8*^-free C57BL/6J mice from the Jackson Laboratory (Bar Harbor, ME, USA, #000664). These crosses produced hemizygous APOB100 Tg mice, which were used in all experiments, and the nontransgenic littermates, served as wild type (WT). Animals were maintained on a standard 12-h light (~ 10 lx)-dark cycle and were provided regular rodent chow and water ad libitum. No statistical methods were used to predetermine sample size, which was based on previous experience. The investigators were not blinded with respect to the mouse genotype. Nevertheless, the quantitative retinal assessments were not affected by investigators’ bias as all data were used and apparent outliers were not excluded. To minimize investigators’ bias in the nonquantitative assessments, which pertained to histochemistry and transmission electron microscopy (TEM), retinal regions that were compared between the genotypes were matched by animal age, sex, and location. All animal experiments were approved by Case Western Reserve University’s IACUC and conformed to recommendations of the American Veterinary Association Panel on Euthanasia (protocol 2014-0154).

### In vivo retinal imaging

Retinal spectral domain optical coherence tomography (SD-OCT) and fundus angiography were carried out as described [38–40]. Envisu R2200 UHR OCT imaging system (an optical axial resolution of 1.93 μm and in tissue refractive index R of 1.38 that is digitized in tissue with a pixel length of 1.65 μm, Leica Bioptigen, Morrisville, NC, USA) and a scanning laser ophthalmoscope (Spectralis HRA, Heidelberg Engineering, Franklin, MA, USA) were used, respectively. Fluorescein angiography (FA) was carried out after a bolus (0.1 ml) intraperitoneal injection of 1.0% sodium fluorescein (Akorn Inc, Lake Forest, IL, USA, #17,478–250-20) in phosphate buffer saline (PBS). Images were taken at ~ 0.75 min (an early phase), ~ 6.5 min (an intermediate phase), and ~ 12 min (a late phase). Indocyanine green (Diagnostic Green, Farmington Hills, MI, USA, NDC 70100-424-01) angiography (ICGA) was performed after a bolus 2 mg/kg of body weight intraperitoneal injection of the dye (~ 0.02 ml of the 2.5 mg/ml ICG solution in sterile water). Images were taken at ~ 3 min (an early phase), ~ 13 min (an intermediate phase), and ~ 25 min (a late phase) at a fixed 100% intensity of brightness with a sensitivity of 90 arbitrary units throughout all phases. The fundus integrated fluorescence intensity was quantified by the Metamorph software (Molecular Devices, LLC, San Jose, CA, USA).

### Lipid histochemistry

This was as described using stains with filipin (Cayman Chemical, Ann Arbor, MI, USA, #70,440) for UC and esterified cholesterol (EC) [38, 41, 42] and BODIPY 493/503 (ThermoFisher Scientific, Inc., Waltham, MA, USA, D3922) for UC, EC, triacylglycerides, and free fatty acids [43].

### Transmission electron microscopy (TEM)

Tissue processing and imaging were as described [44], using the osmium-tannic acid-para-phenylenediamine technique to preserve membranes and neutral lipids [45].

### Serum lipid panel and retinal sterol quantifications

Mice were fasted overnight and deeply sedated the next morning with a 80 mg/kg ketamine and 7 mg/kg xylazine bolus injection (Patterson Veterinary, Greeley, CO, USA, 07-890-8598 and 07-808-1947, respectively). Terminal blood collection was carried out via cardiac puncture followed by retinal (the neural retina plus RPE) isolation as described [39, 46]. Serum was sent to IDEXX Laboratories (North Grafton, MA, USA) for the measurements of total, HDL, and LDL cholesterol, triglycerides, and albumin. Retinal sterols were quantified in the laboratory as described [8, 39] by isotope dilution gas chromatography-mass spectroscopy using individual or pooled samples of mouse retina. The content of both retinal total cholesterol (TC) and UC was measured, thus enabling the calculation of the content of retinal EC.

### Retinal cholesterol input quantifications

These quantifications were previously developed by us for mice and hamsters [36, 37, 47]. First, the rate of total tissue cholesterol input is measured by administering deuterated water (D_2_O) to mice. Then, the rate of tissue uptake of systemic cholesterol is determined in a separate experiment by feeding mice deuterated cholesterol (D_7_-cholesterol). The difference between the two rates is next calculated and represents the rate of tissue cholesterol biosynthesis [36, 37, 47]. To measure total retinal (the neural retina plus RPE) cholesterol input, WT and APOB100 Tg mice were put for 8 weeks on a custom-made fat-enriched (10% peanut oil) and cholesterol-enriched (0.3%) diet (FCED) and received normal drinking water during the first 6 weeks of the FCED administration. Then, mice were injected intraperitoneally with 0.5–0.8 ml D_2_O (equal to ~ 3.5% of mouse body water), and normal water was replaced with 6% D_2_O (v/v) for the next 2 weeks until animals were euthanized. To measure retinal uptake of dietary cholesterol, a separate group of mice was put on normal water and FCED during the first 6 weeks of the experiment. Then the unlabeled cholesterol in FCED was replaced with 0.3% D_7_-cholesterol for the next 2 weeks of treatment until animals were euthanized. Before euthanasia, all animals were fasted overnight and deeply sedated the next morning. Blood was obtained (see previous section), and animals were perfused through the heart with 50 ml of PBS, 1.5 ml/min. Mouse eyes were excised and dissected to obtain the retina, which contained the neural retina and RPE. Subsequent tissue processing and calculations of retinal cholesterol uptake from the systemic circulation and in situ biosynthesis were as described [47]. Deuterium whole-body water enrichment was measured as well as described [36] after the isotopic exchange with acetone of the serum of mice, which received D_2_O.

### Tracking of ICG after in vivo injection

Mice were either non-fasted (for histological ICG tracking) or fasted overnight for plasma LPP isolation. In both cases, mice were injected intraperitoneally with ICG (2 mg/kg of body weight) and 13 min post-injection euthanized. Control animals were injected with sterile water.

For plasma LPP isolation [48], blood samples from 11-month old male mice (four per genotype) were collected via cardiac puncture in EDTA-coated tubes and remained at room temperature for 20 min. Samples were then spun down individually at 2000*g* and 4 °C for 10 min to remove erythrocytes and white blood cells, and the supernatants obtained were combined to have one pooled sample per genotype. These pooled samples were then subjected to centrifugation at 15,000*g* and 4 °C for 30 min to pellet the platelets and float chylomicrons, which were then removed from the top in a 0.1 ml aliquot [49]. The density of the remaining plasma samples (0.93 ml for each genotype) was adjusted to 1.063 g/ml by KBr (Fisher Scientific, Hampton, NH, USA, S80134, 0.0834 g KBr/ml of plasma), the samples were transferred to the bottom of ultracentrifuge tubes, and carefully overlayed with 11 ml of cold solution of KBr of the same density (8.34 g/100 ml) in PBS to fill the tubes. Samples were subjected to ultracentrifugation in the bucket rotor at 100,000*g* and 10 °C for 24 h, and the tube content was then carefully removed in 0.7 ml aliquots from the tube top. The (VLDL + LDL) and HDL-containing fractions were identified at the top and bottom of the tubes, respectively [50], by the measurements of ICG fluorescence at 832 nm (excitation at 720 nm), light scattering (depends on particle concentration and size) at 520 nm, and optical density (proportional to protein concentration) at 280 nm. The density of the HDL-containing fraction was adjusted to 1.21 g/ml by adding KBr (0.235 g/ml), and the fraction was placed at the bottom of the ultracentrifuge tube (1.5 ml). The tubes were filled with 10 ml of cold KBr solution in PBS (31.5 g KBr/100 ml) of the same density and subjected to the 2nd ultracentrifugation at 100,000*g* and 10 °C for 24 h. The tube content was removed in 0.7 ml aliquots and analyzed for ICG fluorescence at 832 nm, light scattering at 520 nm, and optical density at 280 nm to identify the HDL and LPP-free fractions. The LDL and HDL fractions were also analyzed by SDS-PAGE and Western blotting after fraction desalting by dilution (threefold, with 10 mM Tris–HCl, pH 7.4) and concentration 5 times on the 3 KDa cut off filter (ThermoFisher Scientific, Inc., #88512).

For SDS-PAGE and Western blotting of the density ultracentrifugation fractions, 4–15% Tris/Glycine gels (Bio-Rad, Hercules, CA, USA) were used. Proteins after separation were either stained by the Silver Stain Kit (ThermoFisher Scientific Inc., #24612), according to the manufacturer’s instructions, or transferred on the nitrocellulose membranes (Li-Cor Biosciences, Lincoln, NE, USA, P/N 926-31092) followed by membrane blocking in the Odyssey blocking buffer (Li-Cor Biosciences, #927-40000) for 1.5–3 h at room temperature. Subsequent incubations with primary antibody (overnight at 4 °C) and secondary antibody (1 h at room temperature) were in the Odyssey blocking buffer supplemented with 0.1% Tween-20. Primary rabbit monoclonal antibody to APOA1 (Abcam, Cambridge, MA, USA, #ab308187, 1:1000 dilution), rabbit polyclonal antibody to APOA2 (Invitrogen, Waltham, MA, USA, #710260, 1:250 dilution), rabbit polyclonal antibody to APOC2 (Invitrogen, #PA5102480, 1:1,000 dilution), rabbit polyclonal antibody to APOC3 (Invitrogen, #PA5116572, 1:1,000 dilution), and rabbit polyclonal antibody to mouse serum albumin (Abcam, Cambridge, #ab34807, 1:1,000 dilution) were used. The secondary antibody was goat anti-rabbit conjugated to IRDye 800 CW (Li-Cor, Lincoln, #926-68075, 1:25,000 dilution). The intensity of fluorescent bands on the nitrocellulose membranes was quantified by an Odyssey infrared imaging system (Li-Cor Biosciences).

For histological ICG tracking, mouse eyes were enucleated 13 min post ICG injection and fixed for 15 min at room temperature in 20% dimethylsulfoxide (Fisher Scientific, Hampton, NH, USA, BP231-100) plus 2% paraformaldehyde in PBS. Dimethylsulfoxide was used as it is a common cryoprotectant for the sample structural preservation, which impaires ice damage during snap freezing [51, 52]. Eyes were then embedded in the Tissue Tek O.C.T. compound (Sakura Finetek USA, Inc., Torrance, CA, #4583,) and flash frozen in liquid nitrogen. Retinal sections were cut at a 15 μm thickness and imaged on a LSM 980 microscope (Carl Zeiss Microscopy, LLC, White Plains, NY, USA) with an excitation of 730 nm and an emission filter of 755–900 nm. A transmitted-photomultiplier tube at a 488 nm laser excitation was used to acquire the ‘phase like’ images collected at the default settings.

### Evaluation of retinal function

Electroretinography responses **(**ERGs) were assessed as previously described [39] by recording the scotopic a- and b-waves and the photopic b-wave in response to strobe flash stimuli under dark- and light- adapted conditions (scotopic and photopic, respectively).

### Retinal proteomics

The relative protein abundance in the retina was assessed by the label-free approach by Creative Proteomics (Shirley, NY, USA), a proteomics mass spectrometry. Five biological replicates (the neural retina plus RPE) per group were used, each representing a pooled sample of three retinas from three different 7.5-month old female mice. Retinal samples were solubilized in a lysis buffer (8 M Urea, 50 mM Tris–HCl, pH 8.0, 50 mM NaCl, 1 mM DTT, and protease inhibitors) by homogenization in a tissue grinder at 65 Hz for 10 min. The lysate was centrifuged at 12,000 rpm and 4 °C for 15 min, and the supernatant was collected. Sixty five microgram of protein (~ 0.1 ml) from each sample were then sequentially mixed and vortexed with methanol (0.4 ml), chloroform (0.1 ml), and water (0.3 ml), and centrifuged at 6,000 g for 15 min. The top aqueous layer was removed, and methanol (0.4 ml) was added to the remaining sample, which was vortexed and centrifuged at 14,000*g* for 5 min to pellet the protein precipitate. Protein precipitates were dissolved in 2 M urea and reduced with 10 mM DTT at 56 °C for 1 h followed by alkylation with 50 mM iodoacetamide for 60 min at room temperature in the dark. Then, 500 mM ammonium bicarbonate was added to the solution to a final concentration of 50 mM and pH of 7.8. Trypsin (Promega, Madison, WI, USA, #VA9000) in 50 mM ammonium bicarbonate was added to protein solution at a 1:200 ratio and incubated at 37 °C for 15 h. Protein trypsinolysis was quenched with 10% trifluoroacetic acid, and the peptides obtained were desalted on C18 SPE column (ThermoFisher Scientific, Inc.) according to the manufacturer’s protocol. Peptides were then lyophilized by vacuum centrifugation, reconstituted in 0.1% (v/v) formic acid in LC–MS/MS grade water (Sigma-Aldrich, Saint Louis, MO, USA, #1.15333), and quantified by the optical density measurement at 280 nm (NanoDrop Eight Microvolume UV–Vis Spectrophotometer, ThermoFisher Scientific, Inc.). Peptide concentration was normalized to 0.1 μg/μL.

Samples (1 μg of protein) were run on an Ultimate 3000 nano UHPLC system (ThermoFisher Scientific, Inc.) coupled to a Q Exactive HF mass spectrometer (ThermoFisher Scientific, Inc.). Samples were first injected onto a trap column (PepMap C18, 100 Å, 100 μm × 2 cm, 5 μm, ThermoFisher Scientific, Inc.) coupled with an analytical column (PepMap C18, 100 Å, 75 μm × 50 cm, 2 μm, ThermoFisher Scientific, Inc.) at a flow rate of 250 nL/min. The mobile phase A for the UHPLC system was 0.1% formic acid in the LC–MS/MS grade water and the mobile phase B was 0.1% formic acid/80% acetonitrile. The liquid chromatography gradient was run from 2 to 8% buffer B over 3 min, from 8 to 20% buffer B over 50 min, from 20 to 40% buffer B over 43 min, and from 40 to 90% buffer B over 4 min. Gradient elution was held in a 90% elution buffer for the next 5 min before being equilibrated in a 5% elution buffer over 20 min. The mass spectrometer was operated in the data dependent acquisition mode. The full scan was performed between 300 and 1650 m/z at the resolution of 60,000 at 200 m/z and the automatic gain control target for the full scan at 3E6. The MS/MS scan was operated in Top 20 mode using the following settings: the resolution of 15,000 at 200 m/z; automatic gain control target 1E5; maximum injection time 19 ms; normalized collision energy at 28%; isolation window of 1.4 Th; and charge sate exclusion: unassigned, 1, > 6; dynamic exclusion 30 s.

Raw data files were processed with the MaxQuant software (Max Planck Institute of Biochemistry, Martinsried, Germany, version 1.6.1.14), and tandem mass spectra were searched against the UniProt reviewed mouse database. Searches were performed with full tryptic specificity, maximum 2 missed cleavages, precursor ion tolerance of 10 ppm and MS/MS tolerance was 0.5 Da. Carbamidomethylation of cysteine was set as a fixed modification, and methionine oxidation and protein N-terminal acetylation were set as variable modifications. The false discovery rate was set to 1% for both peptide and protein levels, and the minimum required peptide length was set to seven amino acids. Proteins were quantified and normalized using MaxLFQ with a label-free quantification (LFQ) minimum ratio of 1.2. The LFQ intensities were calculated using the match between runs feature, and MS/MS spectra were required for LFQ comparisons. Protein intensity values were normalization with median. Differentially abundant proteins were determined by one way ANOVA at a false discovery rate of 0.05.

### Statistical analyses

All statistical analyses and sample size (n) are indicated in the figure legends. Briefly, all quantitative data, except the ERG responses, represent the mean ± SD; the ERG responses represent the mean ± SEM. The data were analyzed either by one-way or two-way ANOVA, unpaired non-parametric Mann–Whitney test or a two-tailed unpaired Student’s t-test. Statistical significance was defined as *, *P* ≤ 0.05; **, *P* ≤ 0.01; ***, *P* ≤ 0.001. The quantitative studies were not affected by investigators’ bias as all data were used and apparent outliers were not excluded.

## Results

### In vivo retinal imaging

The retina of APOB100 Tg mice did not seem to be previously imaged in vivo [28–30]. Hence, we used SD-OCT to characterize retinal gross structure and two types of angiography, FA and ICGA, for vascular examination. FA assesses retinal vasculature [53, 54], whereas ICGA evaluates the choroid [55–57]. The fluorescence emission maximum of ICG is in the far-red region (~ 832 nm) and therefore enables imaging through the blood and ocular pigments [55–57]. In humans, ICGA can visualize lipid accumulations in BrM as they impede ICG passage into the RPE from the ChC and are presented as hypofluorescent spots in late phase ICGA [58–63]. This is in contrast to the normally homogeneous background fluorescence in the ICGA late phase when the dye crosses BrM and is taken up by the RPE [59, 63–65].

**Fig. 2:**
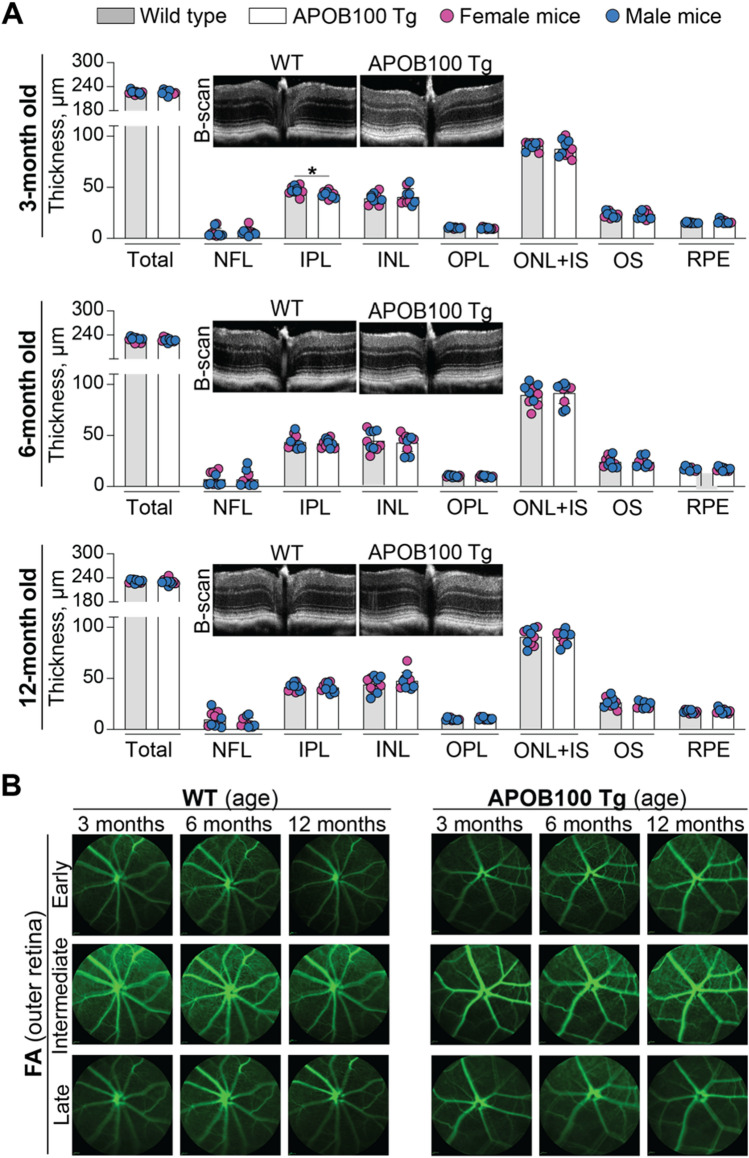
Retinal in vivo imaging in wild type (WT) and APOB100 Tg mice. Animals were monitored longitudinally, i.e., the same animal cohort (5 female and 5 male mice per genotype) was assessed at different ages. **A** Retinal thickness and retinal gross structure (insets) as determined by SD-OCT. Data represent the mean ± SD of the measurements in individual animals after the results from both eyes were averaged. *P ≤ 0.05 as assessed by an unpaired non-parametric Mann–Whitney test. **B** Representative fluorescein angiography (FA) images showing (from top to bottom) an early, intermediate, and late-stage fundus fluorescence. The laser beam was focused on the outer retina. No sex-based differences were detected by either of the imaging modality. The retinal layer labeling is as described in Fig. 1

**Fig. 3:**
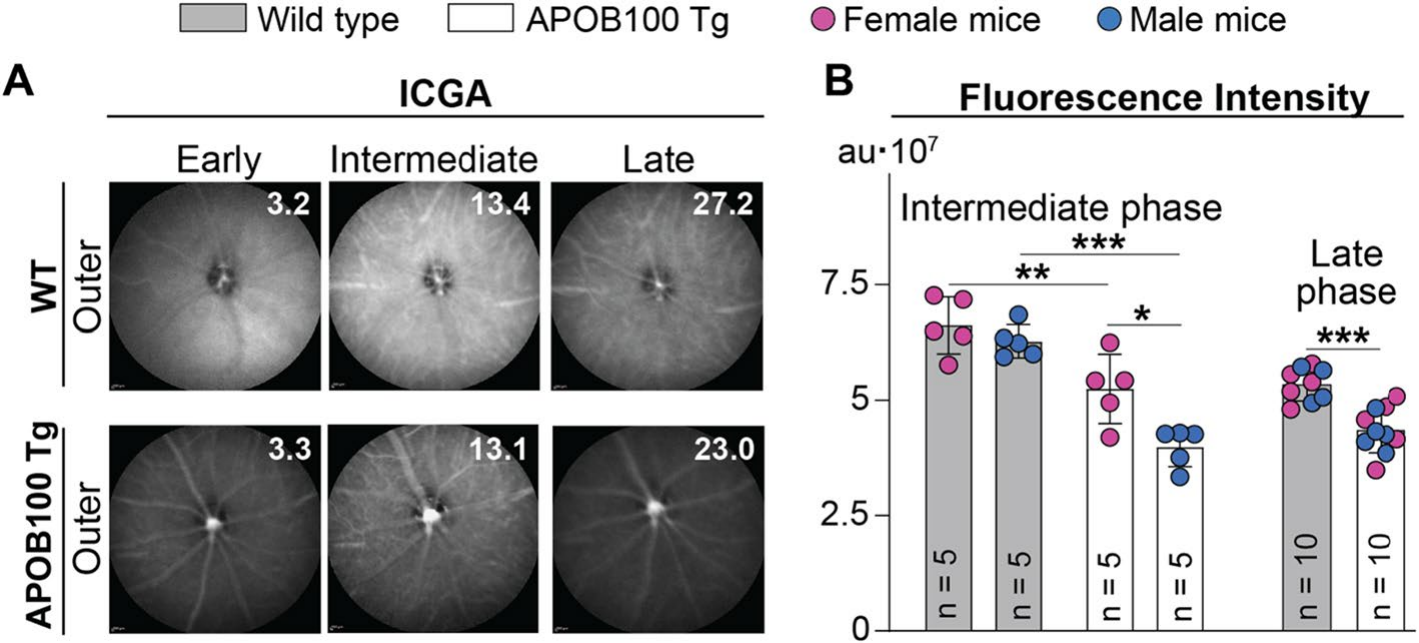
ICG angiography (ICGA) in 1 year old wild type (WT) and APOB100 Tg mice. **A** Representative images (5 female and 5 male mice per genotype) of an early, intermediate, and late-stage fundus ICGA fluorescence with the laser beam being focused on the outer retina, nourished by the choroidal circulation. The precise position of the laser beam cannot be determined, therefore the ICG fluorescence could mainly reflect that in the outer retina but not in the RPE and choroid. White numbers in the upper right corner indicate the post-injection time in minutes. **B** The quantifications of the fundus ICGA fluorescence intensity in (**A**). The number of animals (n) is indicated on each bar; au, arbitrary units. Data were analyzed by two-way ANOVA with Tukey’s multiple comparisons test. When no statistical significance was found between female (magenta circles) and male (blue circles) mice, data for both sexes were combined and assessed for genotype differences by an unpaired t-test; otherwise, data were presented separately. *P ≤ 0.05; **P ≤ 0.01; ***P ≤ 0.001

No changes in retinal gross structure or thickness of retinal layers, including the RPE, were detected on SD-OCT in 3-, 6- and 12-month old APOB100 Tg mice (Fig. 2A). Similarly, FA did not reveal any abnormalities in early, intermediate, and late phases when the laser beam was focused either on the inner (the data not shown) or the outer retina (Fig. 2B). Therefore, ICGA was used only on 12-month old mice and suggested that the overall fundus fluorescence intensity in APOB100 Tg vs WT mice could be lower in the intermediate and late ICGA phases (Fig. 3A). The fluorescence intensity quantifications confirmed this inference and documented that in the intermediate ICGA phase, the integrated fluorescence intensity in female APOB100 Tg vs female WT mice was 5.2**·**10^7^ vs 6.6**·**10^7^ arbitrary units (au), and 4.0**·**10^7^ vs 6.3**·**10^7^ au, respectively, in male mice. In the late ICGA phase, the numbers for mice of both sexes were 4.4**·**10^7^ vs 5.4**·**10^7^ au, respectively (Fig. 3B). Thus, the ICGA quantifications indicated that there may be some differences between APOB100 Tg and WT mice in how the ICG is distributed in the chorioretinal complex either because of lipid accumulation in BrM in APOB100 Tg mice or some other reasons. Hence, next, we used various approaches to further characterize APOB100 Tg mice.

### Chorioretinal lipid distribution

**Fig. 4:**
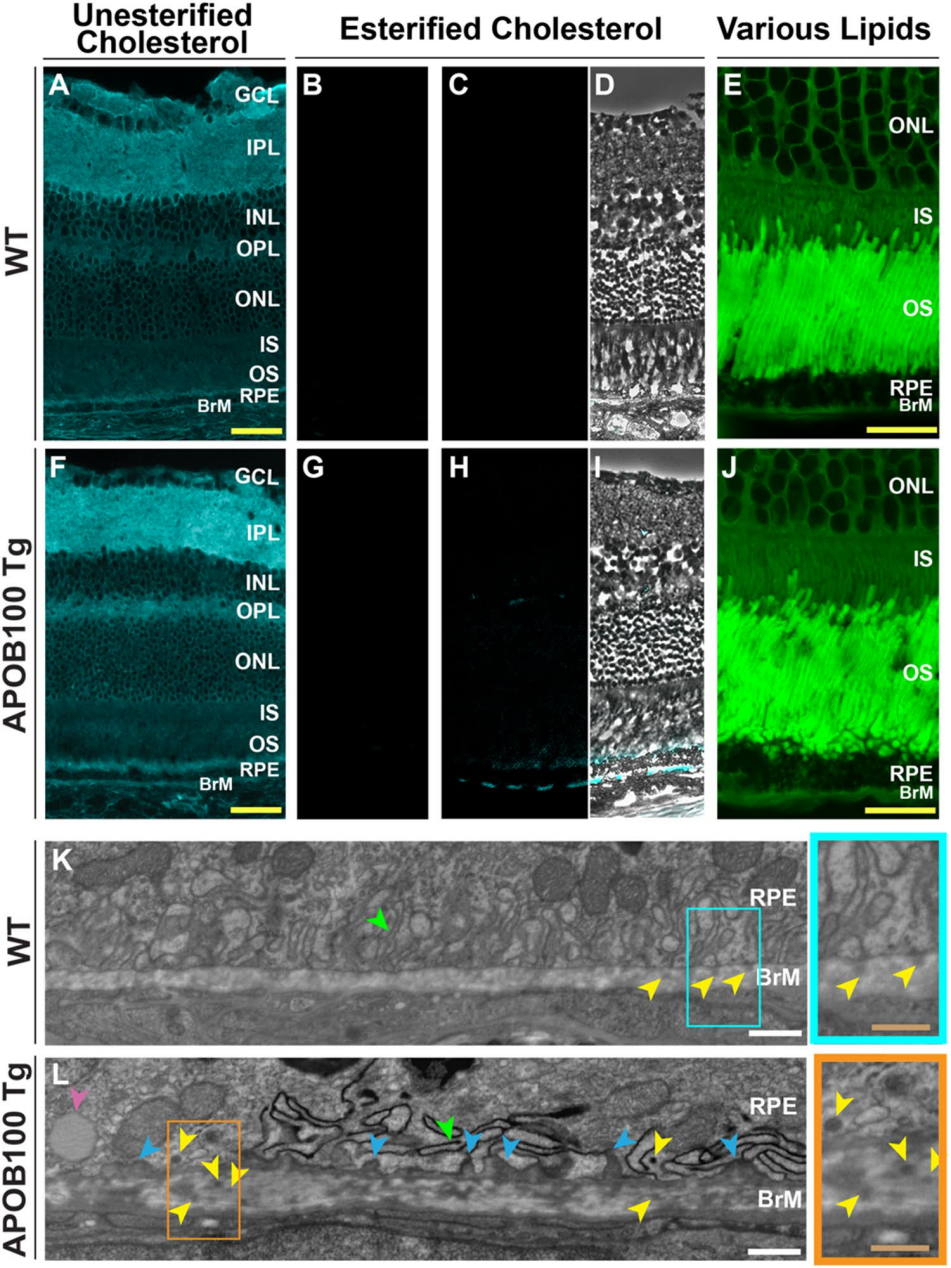
Chorioretinal lipid distribution in wild type (WT) and APOB100 Tg mice. **A**, **F** Representative stains (3 female and 3 male 12-month old mice per genotype) with filipin for unesterified cholesterol. **B**, **G** Control stains for completeness of unesterified cholesterol removal. **C**, **D** and **H**, **I** Representative stains (3 female and 3 male 12-month old mice per genotype) with filipin for esterified cholesterol; **D** and **I** filipin stains for esterified cholesterol overlayed with the phase contrast images to visualize retinal layers. **E**, **J** Representative stains (3 female and 2 male 6-month old male mice per genotype) with BODIPY for unesterified cholesterol, esterified cholesterol, triacylglycerides, and free fatty acids. **K**, **L**, Representative images (1 female and 2 male 12-month old mice per genotype) of tissue ultrastructure as assessed by transmission electron microscopy. Green arrowheads denote basal infoldings; yellow arrowheads point to LPPs, the magenta arrowhead points to a lipid droplet, and blue arrowheads indicate basal laminar deposits. Colored rectangles denote enlarged regions. The retinal layer labeling is as described in Fig. 1. Yellow scale bars are 25 μm, white scale bars are 1 μm, and light brown scale bars are 0.5 μm

We used fluorescent histochemistry stains filipin (without and with additional tissue processing) to label UC and EC, respectively [38, 41, 42] in 12-month old mice, and BODIPY 493/503 to visualize UC, EC, triacylglycerides, and free fatty acids [43] in 6-month old animals. Filipin labeling of UC was very similar between WT and APOB100 Tg mice with perhaps somewhat thicker apical and basal RPE membranes in APOB100 Tg vs WT mice (Fig. 4A, F). Filipin labeling of EC was undetectable in the WT chorioretinal region but was visible in APOB100 Tg mice as linear deposits in BrM and diffuse stain of the RPE apical membrane (Fig. 4C, H). BrM seemed to be thicker in APOB100 Tg vs WT mice with the BODIPY stain (Fig. 4E, J), also suggesting that APOB100 Tg mice could have more lipids in BrM than WT mice.

BrM appeared to be thicker in 12-month old APOB100 Tg vs WT mice when examined by TEM and seemed to contain small particles, which were more electron dense and numerous in APOB100 Tg mice (Fig. 4K, L). These particles were 50–100 nm in diameter and could represent different types of LPPs trafficking to and from the RPE. In addition, the RPE in APOB100 Tg mice had three features that were not detected in WT mice. These were: (1) lipid droplets, usually formed by cholesterol esters; (2) basal laminar deposits (a normal age-dependent deposition of the RPE basement membrane material, which when thick can become specific for AMD [1]); and (3) disturbed basolateral infoldings over basal laminar deposits (Fig. 4K, L). Thus, the RPE-BrM complex ultrastructure was affected by the APOB100 transgenic expression, consistent with the lipid histochemistry findings and previous studies of this genotype [28–30].

### Retinal sterol levels

**Fig. 5:**
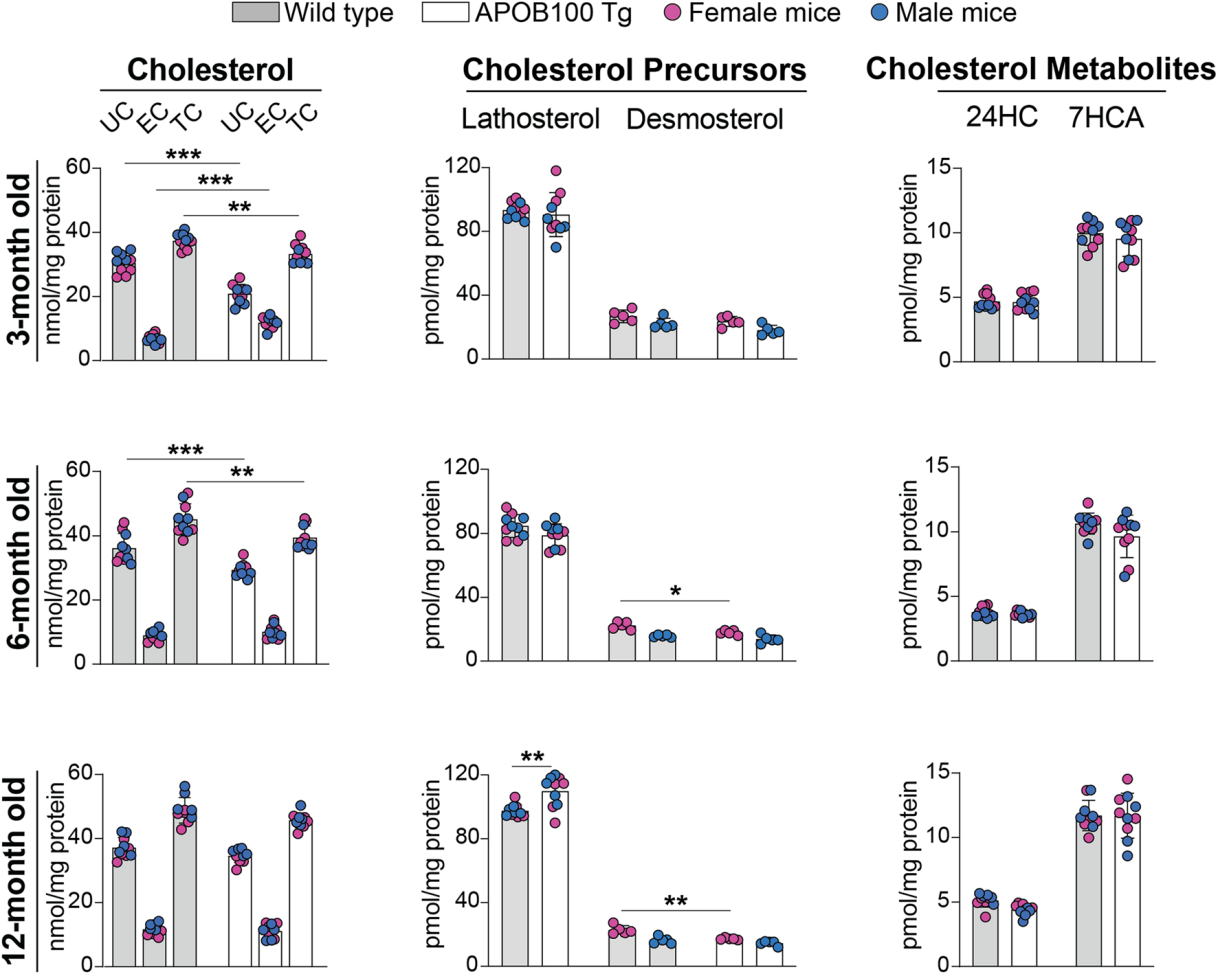
Retinal sterols in wild type and APOB100 Tg mice of different ages. Data represent the mean ± SD of the measurements either in individual retinas [unesterified cholesterol (UC), esterified cholesterol (EC), total cholesterol (TC), lathosterol, and desmosterol: 5 female and 5 male mice per sterol group, genotype, and age] or in pooled samples, each containing 2 retinas from 2 different animals of the same age and sex (all cholesterol metabolites: 5 samples from female and 5 samples from male mice per each metabolite). Data were analyzed by two-way ANOVA with Tukey’s multiple comparisons test. When no statistical significance was found between female (magenta circles) and male (blue circles) mice, data for both sexes were combined within each age, genotype, and sterol group; otherwise, data were presented separately. *P ≤ 0.05; **P ≤ 0.01; ***P ≤ 0.001. 24HC, 24-hydroxycholesterol; 7HCA, 7α-hydroxy-3-oxo-4-cholestenoic acid. 27-Hydroxycholesterol and 5-cholestenoic acid were below the limits of detection (1 pmol/mg protein)

EC deposits in BrM prompted longitudinal (at 3-, 6-, and 12-months of age) quantifications of retinal sterols, which had never been carried out in APOB100 Tg mice (Fig. 5). At 3 months of age, both sexes of APOB100 Tg vs WT mice had a decrease in the UC content (22 vs 31 nmol/mg protein) but an increase in the EC content (12 vs 7 nmol/mg protein), which, however, did not compensate for the UC decrease. Accordingly, the total cholesterol (TC) content was modestly decreased (33 vs 37 nmol/mg protein) as well. Yet, the levels of the biosynthetic cholesterol precursors (lathosterol and desmosterol, markers of cholesterol biosynthesis in neurons and astrocytes, respectively, [37, 66]) as well as cholesterol metabolites (24-hydroxycholesterol and 7α-hydroxy-3-oxo-4-cholestenoic acid, products of enzymatic activities of CYP46A1 and CYP27A1, respectively, [67, 68]) were the same in the two genotypes. At 6 months of age, the levels of UC and TC were also lower in both sexes of APOB100 Tg vs WT mice (29 vs 36 nmol/mg protein for UC and 40 vs 45 nmol/mg protein for TC) but the EC levels became the similar (10 vs 9 nmol/mg protein). In addition, female APOB100 Tg mice had a decrease in the desmosterol levels (18 vs 22 pmol/mg protein). Finally, at 12 months of age, the levels of all forms of cholesterol (UC, EC, and TC) were the same in APOB100 Tg vs WT mice of both sexes, likely because of the compensatory increase in the lathosterol levels in both sexes (110 vs 98 pmol/mg protein), despite a decrease in the desmosterol levels in female APOB100 Tg mice (17 vs 23 pmol/mg protein). Thus, with age, there was a homeostatic response in the APOB100 Tg retina reflected by a decrease in the EC levels and an upregulation of in situ cholesterol biosynthesis. This ultimately led to the normalization of retinal cholesterol levels with no effect on enzymatic cholesterol elimination via the production of oxysterols. As for lipid droplets in the RPE and focal EC deposits in BrM of APOB100 Tg mice (Fig. 4), despite the same retinal EC levels, this could be due to either accumulation of all retinal EC in APOB100 Tg mice in the RPE and BrM or their EC increase at 3 months of age.

### Retinal cholesterol input

**Fig. 6:**
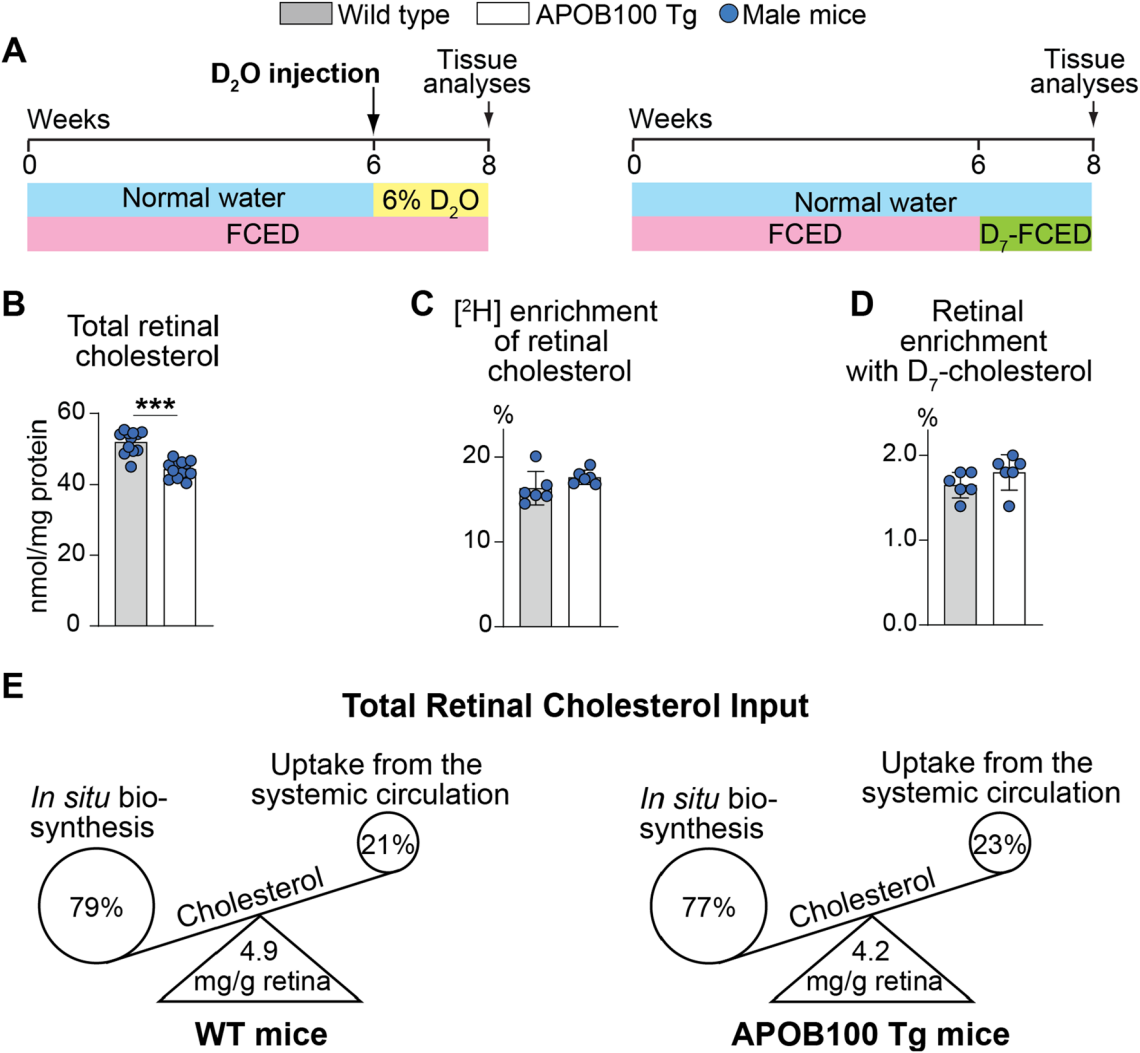
The quantification of the pathways of retinal cholesterol input. Animals were 9.5-month old male mice. **A** Schematic representation of the experiments. FCED, fat- and cholesterol-enriched diet; D_7_-FCED, FCED containing D_7_-cholesterol; D_2_O, deuterated water. **B**–**D** Sterol quantifications. **E** Schematic summary of the results. Data were analyzed by a two-tailed unpaired Student’s t-test (5 male mice per group). ***,* P* ≤ 0.001. WT, wild type

In any tissue, the steady state cholesterol levels represent a balance between the pathways of cholesterol input and output. Since retinal cholesterol output by metabolism to oxysterols was not changed in APOB100 Tg vs WT mice (Fig. 5), we next determined whether the APOB100 Tg expression affected the pathways of retinal (neural retina plus RPE) cholesterol input. We used 9.5-month old animals and first determined the rate of total tissue cholesterol input by administering D_2_O to mice on FCED (Fig. 6A). After 8 weeks on FCED, the levels of total retinal cholesterol were modestly decreased (by 15%) in APOB100 Tg vs WT mice (44 vs 53 nmol/mg protein, Fig. 6B). Yet deuterium [^2^H] enrichment of retinal cholesterol, which reflects total retinal cholesterol input, was similar in both genotypes (16.3% in WT mice and 17.6% in APOB100 Tg mice, Fig. 6C). The rate of tissue uptake of cholesterol from the systemic circulation was next measured by giving mice FCED containing D_7_-cholesterol (Fig. 6A). Like in the measurements of total retinal cholesterol input, essentially no difference was found between APOB100 Tg vs WT mice in retinal enrichment with D_7_-cholesterol (1.81% vs 1.65%, Fig. 6D). The absolute rates of the total retinal cholesterol input and retinal uptake of cholesterol from the systemic circulations were then calculated as well as the difference between the two, representing in situ biosynthesis (Table 1). These calculations revealed that APOB100 Tg vs WT mice had very similar rates of total retinal cholesterol input (0.066 vs 0.070 mg/day/g wet tissue) and uptake by the retina of cholesterol from the systemic circulation (0.015 vs 0.015 mg/day/g wet tissue). Accordingly, the relative contributions of cholesterol uptake from the systemic circulation and in situ biosynthesis were 21% and 79%, respectively, in WT mice and 23% and 77%, respectively, in APOB100 Tg mice, i.e., very similar. Thus, cholesterol input to the retina was not principally unaffected in APOB100 Tg vs WT mice, prompting the measurements of the serum lipid panel and experiments, in which the ICG-bound LPPs were tracked in the plasma and retina after the intraperitoneal dye injection.

**Table 1:** Cholesterol input to the retina of WT and APOB100 Tg mice

Parameter	WT mice		APOB100 Tg mice	
	Serum	Retina	Serum	Retina
Cholesterol concentration: serum (mg/dL); and retina (nmol/mg protein)	111	52	148	44
Cholesterol concentration (mg/g wet tissue)		4.9		4.2
Absolute rate of cholesterol input (mg/day/g wet tissue)		0.070 (100%)		0.066 (100%)
Uptake from blood (mg/day/g wet tissue)		0.015 (21%)		0.015 (23%)
In situ biosynthesis (mg/day/g wet tissue)		0.055 (79%)		0.051 (77%)
Tissue cholesterol turnover (days)		70		64

### Serum lipid profiles

**Fig. 7:**
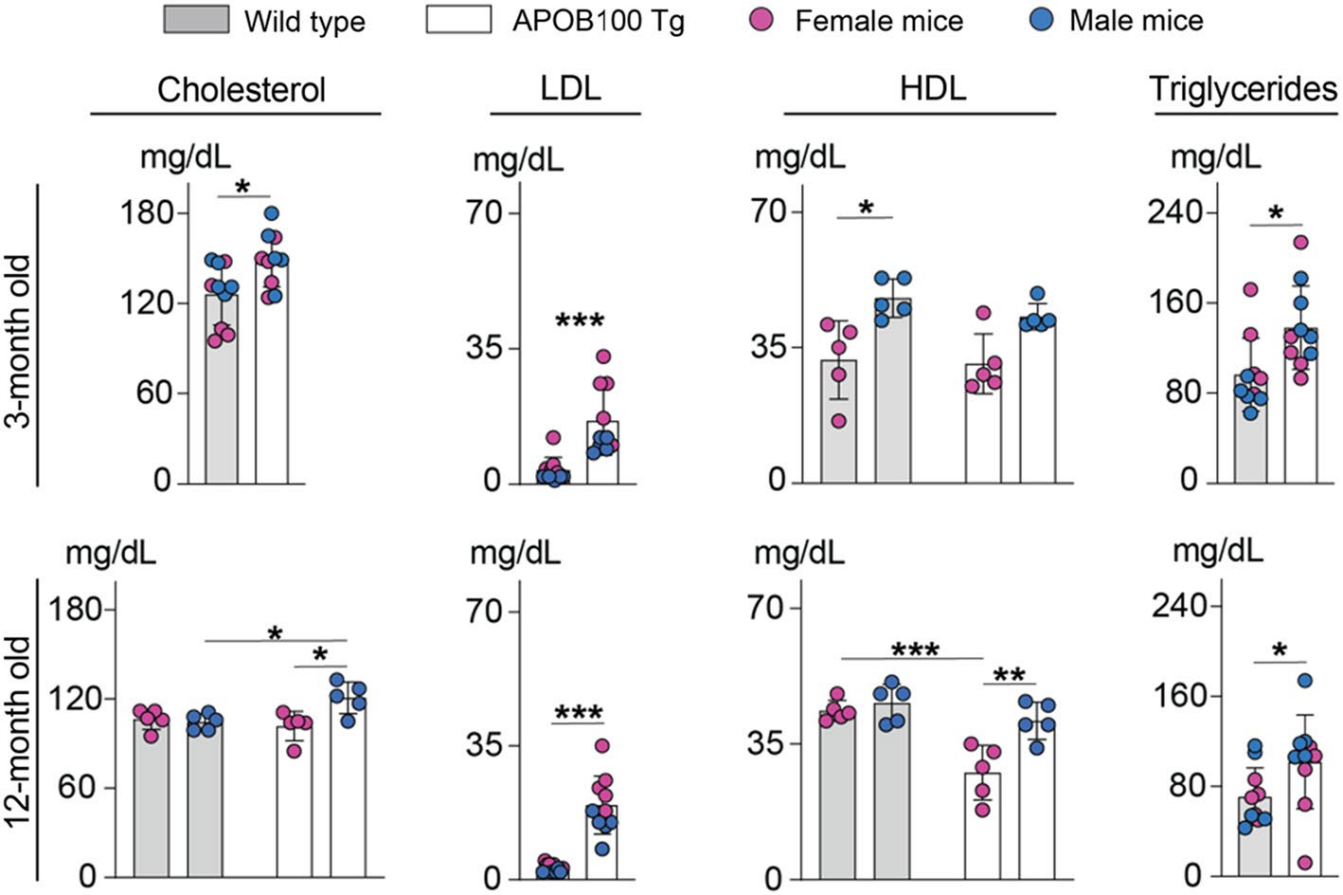
Fasting serum lipid profiles in wild type and APOB100 Tg mice of different ages. Data represent the mean ± SD of the measurements in 5 female (magenta circles) and 5 male mice (blue circles) per each age and genotype group. Data were first assessed for normality of distribution by the Shapiro–Wilk test and then by Kruskal Wallis test for the groups that did not pass the normality test (LDL at all ages, HDL at 3 months of age, and triglycerides at 12 months of age); one-way ANOVA with Tukey’s multiple comparisons test was used for the remaining groups (cholesterol at all ages, triglycerides at 3 months of age, and HDL at 12 months of age). When no statistical significance was found between female and male mice by each test, data for both sexes were combined within each age and genotype group and were assessed for genotype differences either by a parametric unpaired *t* test (for data with normal distribution), or by a non-parametric Mann–Whitney U test (for data, which did not pass the normality test). *P ≤ 0.05; **P ≤ 0.01; ***P ≤ 0.001

Previously, APOB100 transgenic mice were shown to have an increase in their serum LDL content [32, 33], and we documented that this was the case in our colony of animals (Fig. 7). Studies were conducted on fasted mice that were 3- and 12-month old to encompass the age range used in our animal assessments by various approaches. The content of plasma TC was modestly increased in both sexes in APOB100 transgenic vs WT mice at 3 months of age (149 vs 126 mg/dL) and only in male APOB100 Tg mice at 12 months of age (121 vs 105 mg/dL; female mice: 102 vs 106 mg/dl). Yet, at both ages, female and male APOB100 Tg vs WT mice had increased LDL levels (16.3 and 19.5 vs 3.7 and 3.1 mg/dL, respectively). The triglyceride levels were increased as well (138 and 102 vs 96 and 71 mg/dL, respectively). The plasma HDL cholesterol levels were unchanged in both sexes of APOB100 Tg vs WT mice at 3 months of age (female mice: 31 vs 32 mg/dL; male mice 43 vs 48 mg/dL) and decreased in female APOB100 Tg mice at 12 months of age (female mice: 28 vs 44 mg/dL; male mice 41 vs 46 mg/dL). Thus, transgenic APOB100 expression elicited consistent increases in 3- and 12-month old mice of both sexes in plasma LDL and triglyceride levels but did not consistently affect plasma HDL levels. The caveat is that the HDL particles have a high level of structural and compositional heterogeneity and form different subpopulations, depending on the isolation technique [69]. Therefore, we decided to further characterize plasma LPPs and used density ultracentrifugations [70], the gold standard approach for isolation of different LPPs [71].

### ICG binding in the plasma

**Fig. 8:**
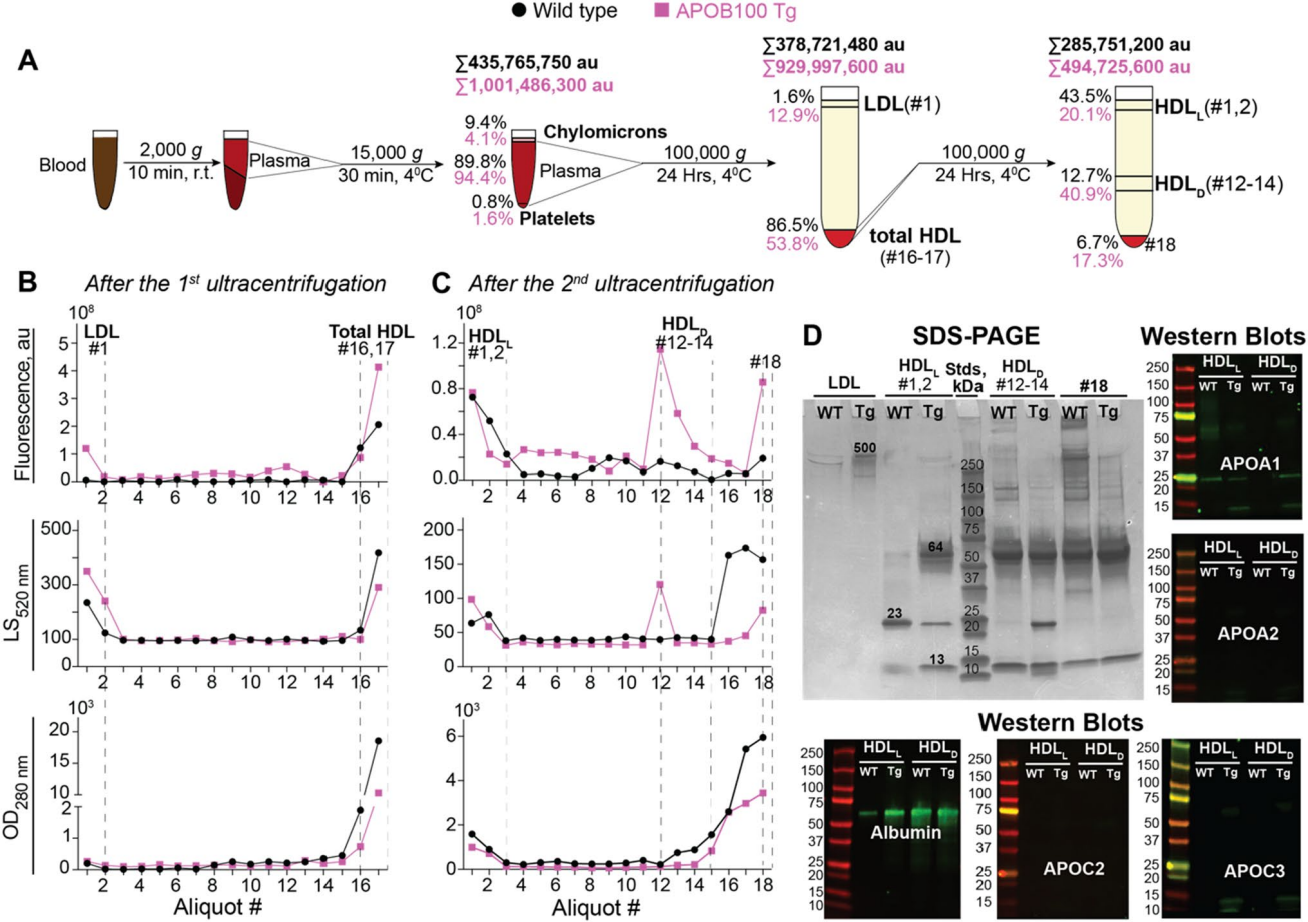
ICG binding in mouse plasma. **A** Schematic representation of the LDL and HDL isolation. Blood samples were collected from 11-month old male mice (four per genotype) and pooled after plasma isolation. The total tube fluorescence and the fluorescence percent in different fractions are also indicated. **B**, **C** ICG fluorescence intensity, light scattering (LS) at 520 nm, and protein optical density (OD) at 280 nm of the aliquots obtained after the 1st and 2nd density ultracentrifugation’s, respectively. Aliquots were collected from the tube top to the tube bottom. **D** SDS PAGE of selected aliquots and Western blots for APOA1, APOA2, APOC1, APOC2, APOC3, and serum albumin of HDL_L_ and HDL_D_. The same amount of protein (12 μg) was applied per each lane for SDS-PAGE. The protein amount per lane for Western blots was as follows: 2.5 μg to detect APOA1, APOA2, APOC2, and APOC3, and 0.2 μg to detect albumin. au, arbitrary units; Stds, molecular weight standards, Tg, APOB100 Tg mice, WT, wild type

We capitalized on earlier investigations showing that ICG interacts with different plasma proteins as well as LDL and HDL, likely because of binding on the latter two to their polar lipids (phospholipids) but not neutral lipids (EC, UC, or triglycerides) [72–76]. Therefore, we injected 12-month old mice intraperitoneally with ICG and 13 min post injection withdrew animal blood. We used two centrifugations to isolate plasma, chylomicrons, and platelets, and then two density ultracentrifugations to isolate the fractions of LDL and HDL (Fig. 8A) [50]. We monitored the ICG fluorescence and in addition, light scattering at 520 nm and optical density at 280 nm (Fig. 8B, C). The latter two distinguish between LPPs that scatter light at 520 nm from plasma proteins and compounds with conjugated double bonds that absorb light at 280 nm but do not scatter it at 520 nm. LPPs were also analyzed by SDS-PAGE and Western blotting for protein composition (Fig. 8D).

The fluorescence measurements revealed that after each centrifugation/ultracentrifugation step, the total tube ICG fluorescence was always higher in the APOB100 Tg vs WT sample: 1,002**·**10^6^ vs 436**·**10^6^ au after the 15,000 g centrifugation; 930**·**10^6^ vs 379**·**10^6^ au after the 1st density ultracentrifugation, and 495**·**10^6^ vs 286**·**10^6^ au after the 2nd density ultracentrifugation (Fig. 8A). With respect to fractions, chylomicrons and platelets collectively had 57**·**10^6^ and 44**·**10^6^ au of fluorescence in the APOB100 Tg and WT samples, respectively (Fig. 8A), and the LDL fraction (aliquot 1, Fig. 8B) contained 120**·**10^6^ and 6**·**10^6^ au of fluorescence in APOB100 Tg and WT mice, respectively. This increase in fluorescence was consistent with more than a sixfold increase in the plasma LDL content in APOB100 Tg mice (Fig. 7) and a higher peak of the LDL fraction light scattering (aliquot 1, Fig. 8B), a marker of LDL concentration.

The ICG fluorescence of the total HDL fraction (aliquots 16 and 17 after the 1st density ultracentrifugation, Fig. 8B) was also higher in APOB100 Tg vs WT mice (500**·**10^6^ vs 328**·**10^6^ au, respectively). Yet the light scattering and protein optical density of this fraction were lower in APOB100 Tg vs WT samples (Fig. 8B), indicating differences in protein and LPP concentration or composition. These differences were then confirmed by the 2nd density ultracentrifugation of the total HDL fraction and the profiles of the ICG fluorescence and light scattering. In particular, in APOB100 Tg vs WT mice, the floating HDL fraction (aliquots 1 and 2, designated as light HDL, HDL_L_, Fig. 8C) had a lower fluorescence (99**·**10^6^ vs 124**·**10^6^ au of fluorescence, respectively). Yet, the APOB100 Tg vs WT genotype had an additional peak of fluorescence (203**·**10^6^ vs 36**·**10^6^ au, respectively) and light scattering in aliquots 12–14 designated as dense HDL, HDL_D_ (Fig. 8C). There was also a higher fluorescence in aliquots 4–11 (Fig. 8C) and in the LPP-free fraction (aliquot 18, Fig. 8C) in APOB100 Tg vs WT mice (86**·**10^6^ vs 19**·**10^6^ au, respectively). However, the light scattering and protein optical density of the LPP-free fraction were lower in APOB100 Tg than WT mice (Fig. 8C). Apparently, increased LDL content, additional HDL_D_ subpopulation, and the LPP-free fraction contributed, at least in part, to increased plasma ICG fluorescence in APOB100 Tg vs WT mice.

SDS-PAGE (Fig. 8D) showed that in both genotypes, the LDL fraction (aliquot 1, Fig. 8B) contained very little protein, consistent with low protein content in this LPP class [77]. Nevertheless, APOB100 (~ 500 kDa), a marker protein for LDL (Fig. 8D), was detectable in APOB100 Tg mice as the major protein band.

The protein composition on SDS-PAGE of the HDL subpopulations was different. In WT mice, the HDL_L_ subpopulation (aliquots 1–2, Fig. 8C) had a prominent band at ~ 23 kDa (immunoreactive for APOA1, a marker protein for HDL) and two less intense bands at ~ 13 kDa and ~ 11 kDa, which were below the reliable detection by Western blotting (Fig. 8D). In contrast, in APOB100 Tg mice, the 23 kDa band in the HDL_L_ subpopulation was not as strong and had a comparable intensity with the ~ 13 kDa band (immunoreactive for APOC3, a component of HDL) and the 64 kDa band immunoreactive for albumin (Fig. 8D). Notably, in both genotypes, the 64 kDa band was prominent in all aliquots after the 2nd ultracentrifugation, except the HDL_L_ subpopulation in WT mice.

The HDL_D_ subpopulation (aliquots 12–14, Fig. 8C) was mainly detected in APOB100 Tg mice based on peaks of ICG fluorescence and light scattering, which were absent in WT mice (Fig. 8C). This subpopulation had only APOC3 (the 13 kDa band) as the major apolipoprotein on SDS-PAGE in WT mice. Yet in APOB100 Tg mice, the HDL_D_ subpopulation also had APOA1 (the 23 kDa band), which was of a similar intensity as APOC3 (Fig. 8D). Finally, the 13 kDa band was strong in the LPP-free fractions of both genotypes, which also contained a lot of other proteins with the most prominent protein being albumin (the 64 kDa band). Thus, APOB100 Tg and WT mice had different protein levels and composition in each tested fraction, and the HDL fraction was mainly represented by the two subpopulations, HDL_L_ and HDL_D_, in APOB100 Tg mice and one subpopulation, HDL_L_, in WT mice.

Since the HDL_L_ subpopulation in APOB100 Tg mice seemed to have increased albumin content as compared to WT mice (Fig. 8D), we decided to ascertain whether this increase reflects an increase in the total serum albumin content. We used a separate group of mice, whose serum was not fractionated, and established that the total serum albumin content was actually modestly decreased (by 9%) in APOB100 Tg vs WT mice (2.9 ± 0.1 vs 3.2 ± 0.1 g/dL, *P* ≤ 0.01, n = 4, 9-month old male mice). Thus, APOB100 Tg expression seemed to increase the albumin content in the plasma HDL_L_ subpopulation likely because of a lower albumin content in other LPP or non-LPP fractions, i.e., led to albumin redistribution between different density ultracentrifugation fractions.

### Histological ICG tracking

**Fig. 9:**
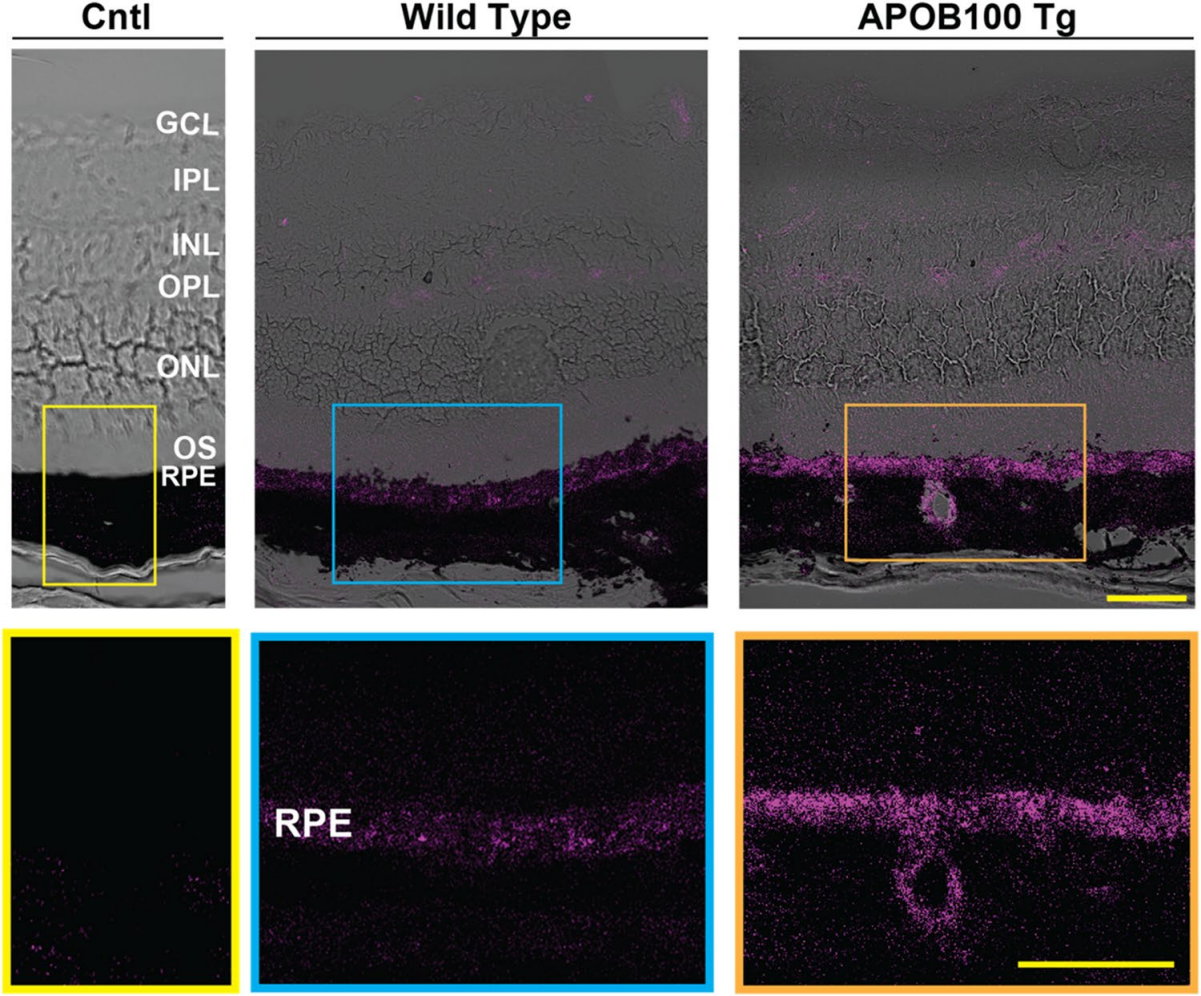
Histological ICG tracking. Representative retinal cross-sections from the eyes of 1 year old wild type and APOB100 Tg mice (2 female mice and one male mouse per genotype) after animals were euthanized 13 min post intraperitoneal ICG injection. Control mouse (Cntl) was injected with sterile water. Color boxes indicate enlarged chorioretinal regions shown at the bottom. The retinal layer labeling is as described in Fig. 1. Scale bars are 50 μm

We investigated whether ICG binding to plasma LPPs could be used a tool to track LPP trafficking from the choroidal and retinal circulations. Mice were intraperitoneally injected with ICG and 13 min later euthanized. Their eyes were enucleated, and retinal cross sections were cut and examined for ICG fluorescence. As compared to angiography, the ICG fluorescence in histological sections is not usually “quenched” and was previously used as an approach complimentary to ICGA [59]. Essentially no fluorescence was observed in control retinal cross sections when sterile water was injected (Fig. 9), yet the fluorescence was visible in the retina of the ICG-injected animals and appeared to be higher in APOB 100 Tg than in WT mice, consistent with their higher plasma ICG fluorescence (Fig. 8A). In both genotypes, the ICG fluorescence was faint around retinal blood vessels (Fig. 9), indicating only minor ICG extravasation into the neural retina at the time point when the ICG fluorescence was the highest on ICGA (Fig. 3). This extravasation could reflect some ICG leakage from the retinal vasculature [78] (either as a free molecule or as bound to a plasma protein) as the inner blood-retinal barrier does not seem to be permeable to plasma LPPs [79]. Alternatively, ICG that leaked from retinal vasculature could label HDL-like particles, which were suggested to be present in the neural retina [5], consistent with the punctate nature of ICG fluorescence in the neural retina.

In both genotypes, most of the ICG fluorescence was accumulated in the RPE and, this fluorescence was also punctate, suggestive of LPP labeling. Similar to the neural retina, the fluorescence intensity seemed to be higher in APOB100 Tg mice than in WT mice. In addition, APOB100 Tg mice but not WT mice had an ICG extravasation in the choroidal blood vessel wall and extravascular choroidal stroma and a continuous track of fluorescent dots connecting the vascular wall and the RPE (Fig. 9). This continuous fluorescence track was consistent with the LPP cycling between the ChC and RPE established previously [12–15] and could represent a more intense cycling process in APOB 100 Tg mice vs WT mice. The latter is supported by data suggesting more than a sixfold increase in the plasma LDL level in APOB100 Tg mice (Fig. 7), numerous LPPs in the RPE-BrM complex detected on TEM (Fig. 4K, L), and a totally different pattern of labeling with filipin and BODIPY (Fig. 4A–J), indictive of ICG binding to the lipids in the structures different from those that have lipids labeled by filipin and BODIPY.

Of importance was that both genotypes had a faint ICG fluorescence in the subretinal space near the RPE, indicating only a moderate LPP release from the RPE apically into the neural retina. This fluorescence pattern was consistent with unchanged retinal cholesterol input in APOB100 Tg vs WT mice (Fig. 6) and in situ biosynthesis being the major source of cholesterol for mouse retina [36, 37]. Thus, combined with the cholesterol input measurements (Fig. 6), histological ICG tracking suggested that the plasma LDL load was handled mainly in the RPE, even when this load was increased > sixfold in APOB100 Tg mice. Increased BrM-RPE LPP cycling correlated with increased albumin content in the plasma HDL_L_ subpopulation and could be a potential homeostatic mechanism to tolerate the increased plasma lipid load.

### Retinal function

**Fig. 10:**
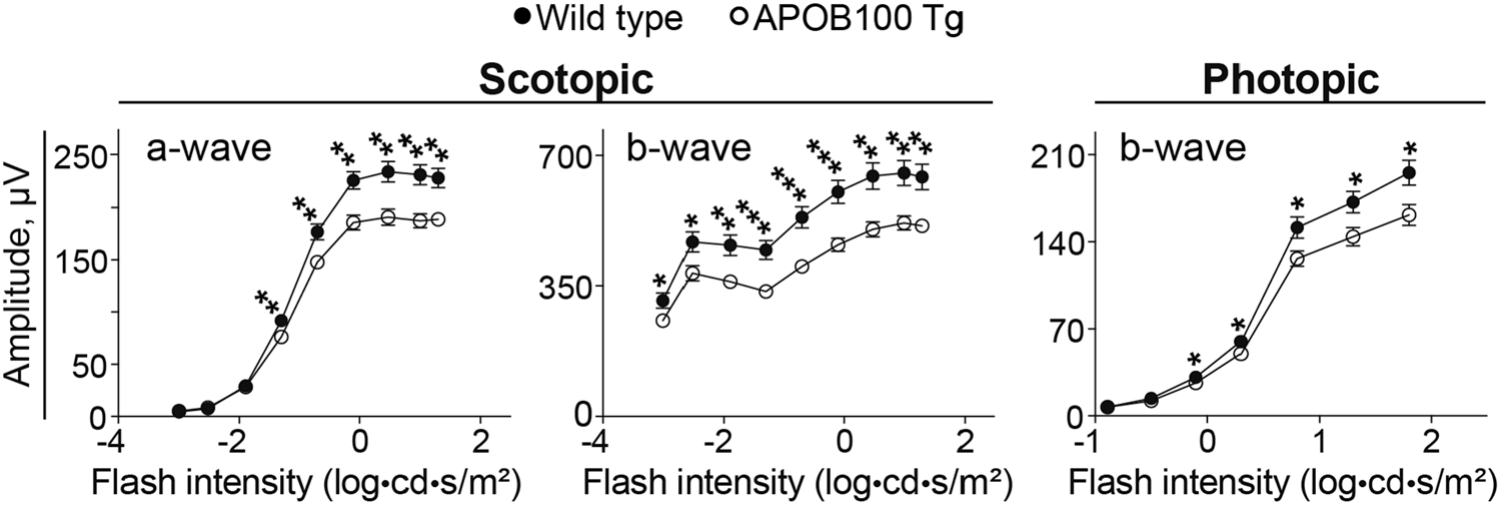
Electroretinography responses in 1 year old wild type and APOB100 Tg mice. Data represent the mean ± SEM of the sum of measurements in both mouse eyes (5 female and 5 male wild type mice and 5 female and 6 male APOB100 Tg mice). Data were analyzed by repeated measured two-way ANOVA. No sex-based difference was found within each genotype. *P ≤ 0.05; **P ≤ 0.01; ***P ≤ 0.001

We recorded ERGs in 12-month old animals as the overall retinal function did not seem to be assessed previously in APOB100 Tg mice [28–30]. The amplitudes of both scotopic (dark-adapted conditions) and photopic (light-adapted conditions) ERG waves were decreased in APOB100 Tg vs WT mice indicating an impairment in the overall retinal function (Fig. 10). This result prompted studies of retinal proteomics to gain unbiassed insights into the processes in the retina that could be affected by APOB100 transgenic expression.

### Retinal proteomics

**Fig. 11:**
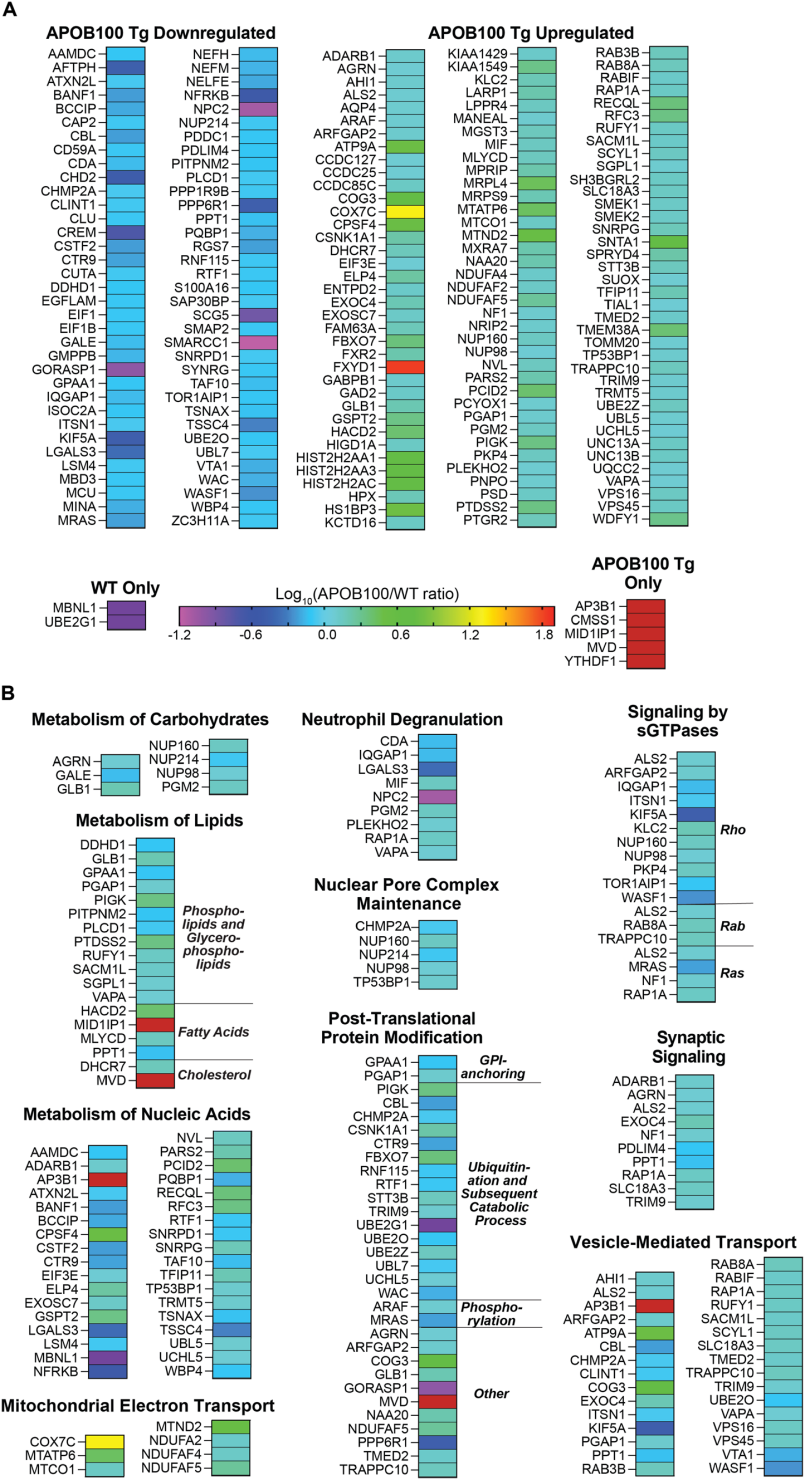
Retinal proteomics of wild type (WT) and APOB100 Tg mice. **A** Differentially expressed proteins. **B** Biological processes enriched with differentially expressed proteins. Five biological replicates per genotype were used, each representing a pooled sample of three retinas from three different 7.5-month old female mice

Changes in retinal protein abundance were assessed by the label free approach, and a total of 4,623 proteins were identified. Of them, 189 were the differentially expressed proteins (DEPs, ≥ 1.2-fold change, an arbitrary cut off) in APOB100 Tg vs WT mice: 72 had decreased expression and 117 had increased expression (Fig. 11A). The DEPs were analyzed for statistical over-representation in the biological processes by the PANTHER software [80], and several of such processes were identified. These were: metabolism of lipids (phospholipids, glycerophospholipids, fatty acids, and cholesterol), carbohydrates, and nucleic acids; neutrophil degranulation; nuclear pore complex formation; post-translation protein modification (GPI-anchoring, ubiquitination, phosphorylation, and other); signaling by sGTPases (Rho, Rab, and Ras); synaptic signaling; vesicle-mediated transport, and TCA cycle along with respiratory electron transport (Fig. 11B).

In addition to DEPs, we also analyzed the relative abundance of the apolipoproteins detected in APOB100 Tg and WT mice (Table 2). APOA1 and APOO were the most abundant apolipoproteins in both genotypes, and abundance of the latter was decreased in APOB100 Tg mice at the level of a trend (*P* = 0.08). APOJ was less abundant than APOA1 and APOO and also had a decreased expression in APOB100 Tg mice (indicated as CLU in Fig. 11). The relative abundance of APOE in both genotypes was lower than that of APOJ, and the abundance of mouse APOB was the smallest. Human APOB was not detected, consistent with the rapid RPE-ChC cycling of the human APOB-containing LPPs. Thus, retinal proteomics suggested a number of biological processes (pathways) that could be affected in APOB100 Tg mice and identified for the first time APOO in mouse retina.

**Table 2:** Relative apolipoprotein abundance (in alphabetical order) in the retina of WT and APOB100 Tg mice as assessed by label free quantifications

Apolipoprotein	Relative abundance in WT mice, au	Relative abundance in APOB100 Tg mice, au	APOB100 Tg/WT, abundance fold change	*P* value
APOA1	431,290,154	487,564,235	1.13	0.4
APOB (mouse)	37,091,637	38,187,411	1.03	0.87
APOB (human)	Not detected	Not detected	Not applicable	Not applicable
APOJ	338,492,190	272,372,919	0.80	0.007
APOE	165,014,676	165,014,676	1.17	0.23
APOO	448,059,975	330,265,252	0.74	0.08

au, arbitrary units

## Discussion

The present work is a continuation of studies in this laboratory aimed at understanding retinal cholesterol homeostasis and whether plasma lipids affect this process. Herein, we characterized APOB100 Tg mice and obtained several novel insights. First, besides a higher plasma LDL content, APOB100 Tg vs WT mice had an additional, higher density HDL_D_ subpopulation, and increased albumin content in the lower density HDL_L_ subpopulation (Figs. 7, 8). Second, ICG fluorescence of retinal cross sections provided a visual proof of LPP cycling between the ChC and RPE and that this cycling could be increased in APOB100 Tg mice (Fig. 9). Third, our data suggested only limited LPP trafficking from the RPE to the neural retina, thus offering an explanation of why in situ biosynthesis is the major source of cholesterol for the retina plus RPE (Fig. 6). Fourth, we discovered that despite altered plasma LPP profile, the quantitative contributions of the pathways of retinal cholesterol input as well as output by metabolism to oxysterols were not altered in APOB100 Tg mice. Fifth, we found that retinal function is impaired in APOB100 Tg mice. Finally, the retinal proteomics data identified biological processes that could be affected by the APOB100 transgenic expression.

Previously, we established that in mice, only 22–28% of total retinal cholesterol input is provided by the systemic circulation [36, 37]. Yet, the plasma LDL content is very low in mice and their plasma LDL/HDL ratio is only ~ 0.07 (Fig. 7) as compared to 2.5–3.0 in healthy humans [81]. Therefore, in another study, we quantified retinal cholesterol input in hamsters, whose whole body cholesterol maintenance is more similar to that in humans than in mice with a higher plasma LDL/HDL ratio of 0.4–0.5 [47]. Hamsters were found to have a higher retinal cholesterol uptake from the systemic circulation than mice (~ 47% of total retinal cholesterol input), thus suggesting that the LDL/HDL ratio could indeed affect retinal cholesterol input. This finding gave impetus to the present work as the APOB100 transgenic expression is known to increase plasma LDL content [32, 33]. We documented that the fasting LDL levels were increased > sixfold in APOB100 Tg mice and that their fasting LDL/HDL ratio was 0.44–0.57 (~ 0.07 in WT mice, Fig. 7), similar to that in hamsters (0.4–0.5) [47], although the absolute LPP levels in APOB100 Tg mice were about 3-times lower than those in hamsters [47]. Herein, we found that both, uptake from the systemic circulation and in situ biosynthesis, were not altered in APOB100 Tg vs WT mice (Fig. 6, Table 1), thus indicating that not only the LDL/HDL ratio, but also other factors likely affect retinal cholesterol input.

One of these factors could be the HDL heterogeneity and an additional HDL density subpopulation detected in APOB100 Tg mice. Based on density ultracentrifugation, HDL is classically divided into HDL_2_ particles, which are large and light, and HDL_3_ particles, which are small and dense [71]. HDL_3_ carries less EC and UC by weight % than HDL_2_ [77, 82] and appears to be functionally superior to HDL_2_ in the ability to promote cellular cholesterol efflux via the ABCA1/G1 transporters, protect LDL from oxidation as well as to inhibit thrombosis, inflammation, and apoptosis [83, 84]. Therefore, despite conflicting clinical data, HDL_3_ seems to be more tightly linked to atheroprotection and clinical cardiovascular disease outcomes than HDL_2_ [85]. Another factor, also of relevance to HDL, could be the serum albumin content, which was shown to facilitate cellular UC efflux to extracellular acceptors (e.g. HDL and LDL) and therefore to be inversely associated with cardiovascular disease [86–89].

We did not characterize the function of HDL_L_ and HDL_D_ isolated from APOB100 Tg mice in the present work, and therefore cannot define them with certainty as HDL_2_ and HDL_3,_ respectively, especially the latter. Nevertheless, it is feasible that a change in the plasma HDL subpopulations in APOB100 Tg mice represented a compensatory response to an increase in their plasma LDL content. If so, the properties of HDL_3_ (see above) suggest that increased HDL_D_ content (putative HDL_3_) and/or increased albumin content in the HDL_L_ (putative HDL_2_) fraction could lead to the following events in APOB100 Tg mice (Fig. 1B). First, a decrease in the EC delivery to the RPE on plasma HDL and subsequent EC uptake by the RPE via SR-BI. Second, a change in the SR-BI-mediated bi-directional UC exchange between the RPE and plasma HDL (as well as LDL [90]). Third, an increase in the UC efflux from the RPE to plasma HDL via ABCA1/ABCG1. In addition, there was likely an increase in the basolateral cholesterol offload on BrM LPPs as suggested by TEM and ICG histology tracking (Figs. 4, 9). As a result, the LPP cycling between the RPE and ChC likely increased (Fig. 9), thus allowing the total retinal cholesterol input to remain unchanged (Fig. 6). In turn, increased LPP cycling probably led to LPP retention and lipid deposition in BrM (Fig. 4) as with age, hydraulic conductivity and permeability to solutes and macromolecules is reduced in BrM, thereby increasing the LPP retention in this layer [91]. However, this lipid deposition did not lead to basal linear deposits or drusen in APOB100 Tg mice, possibly due to an initial decrease in the absolute retinal cholesterol levels (Fig. 5).

Of importance, is that the UC, EC and TC levels were lowered in APOB100 Tg vs WT mice at 3 months of age. This could be due to increased cholesterol offload from the RPE to the choroidal circulation in response to the change in serum lipid profile, thus decreasing the blood-borne cholesterol supply from the RPE to the neural retina. The latter in turn can trigger another compensatory response—an increase in cholesterol biosynthesis in the neurons of the retina as exemplified by increased lathosterol levels in 12-month-old APOB100 Tg mice of both sexes (Fig. 5), and a decrease in cholesterol biosynthesis in retinal astrocytes suggested by decreased desmosterol levels in 12-month old APOB100 Tg female mice. The net result of these and perhaps other (to be identified) compensatory responses in 9.5–12-month old APOB100 Tg mice was essentially unchanged cholesterol uptake by the RPE from the systemic circulation and unchanged in situ biosynthesis in the neural retinal plus RPE (Fig. 6). Obviously, further studies are necessary to support our interpretations and that in humans, a change in the LDL levels and density HDL subpopulations could lead to such a complex series of retinal and RPE responses and contribute to the lack of consistent correlations between plasma lipid profile and AMD [81, 92].

The suggested change in the density HDL subpopulations is supported by the identification of the variants in the genes involved in HDL metabolism (*CETP*, *LIPC*, and *ABCA1*) as risk factors for AMD [93]. Yet, unresolved discordance is that the AMD-associated *CETP* variant increases plasma HDL levels, whereas two AMD-associated *LIPC* variants decrease plasma HDL levels [81]. These data as well as studies showing that increased HDL levels might confer an increased risk of AMD [94, 95] led to a notion in the field that analyses of the HDL-AMD association should not only consider the total HDL levels but also the levels of different HDL subpopulations and their functionality [81, 92]. The present work provides experimental support for this notion and exemplifies how changes in the HDL subpopulations might affect retinal cholesterol maintenance and lead to lipid deposition in BrM. Moreover, studies suggest that even dysfunctional and pro-atherogenic HDL subpopulation could be produced under certain pathologic conditions [84].

ChC accounts for 85% of the blood supply to the retina and its vessels have fenestrations that facilitate fluid, protein, and lipid exchange with the retina [3]. The RPE has different types of receptors on the basal surface that mediate cholesterol uptake and exchange with circulating LPPs [4–7]. Plus, with age, tight junctions of the endothelial cells of the choriocapillaris could become leaky, an additional transport mechanism from the ChC to the RPE [96]. Nevertheless, despite such a favorable arrangement for essentially unlimited cholesterol supply to the retina, cholesterol input from the systemic circulation accounts for less than a half of the total retinal cholesterol input in mice and hamsters (22–28% and 47%, respectively) [36, 37, 47]. These findings raise a question of why more than a half of cholesterol is synthesized in the retina locally? The present study offers an answer to this question by revealing only a moderate apical release of the ICG-bound material (presumably of systemic origin) from the RPE to the neural retina (Fig. 9). In addition, examination by TEM showed that the RPE even begins to store cholesterol excess in the form of lipid droplets, when the plasma lipid profile is changed (Fig. 4). Thus, the RPE seems to limit cholesterol input from the systemic circulation to the neural retina so that the neural retina can synthesize cholesterol locally. Apparently, this allows the neural retina to better control its cholesterol levels and support the needs of synaptic transmission, which depends on cholesterol [97–103], as well as other retinal functions. In this respect the neural retina could be similar to the brain, also a neural organ, in which almost all of its cholesterol is synthesized locally [104].

Retinal proteomics of APOB100 Tg vs WT mice suggested that DEPs were overrepresented in the pathways of cholesterol metabolism (biosynthesis), synaptic signaling, and vesicle-mediated transport (Fig. 11), consistent with changes in retinal cholesterol maintenance (Fig. 5), retinal function (Fig. 10), and increased LPP cycling between the RPE and ChC (Fig. 9). In addition, other pathways could be affected (Fig. 11), although it is difficult to predict from the DEP overrepresentation the overall effect on the pathway function. Nevertheless, altered abundance of specific proteins could be informative and provide mechanistic insights.

For example, MVD (diphosphomevalonate decarboxylase) was detected only in APOB100 Tg mice (Fig. 11). This enzyme catalyzes an important regulatory step in the mevalonate portion of the cholesterol biosynthesis pathway that produces isoprenoids [105], molecules necessary for protein prenylation, a post-translational modification. Notably, prenylation is required for membrane binding and activation of downstream effectors of many members from the Ras, Rho, and Rab families of small GTPases (sGTPases) [106]. Rab proteins are regulators of vesicle trafficking, a process by which newly synthesized transmembrane proteins are delivered from the endoplasmic reticulum via the Golgi to the plasma membranes, whereas some cell-surface proteins (e.g., receptors for extracellular ligands such as LDL) undergo endocytosis and are recycled back to the plasma membranes [106, 107]. Rho and Ras proteins can also affect vesicle transport as they are required for the reorganization of actin cytoskeleton, an essential contributor to intracellular vesicle transport [108]. In addition, Rho and Ras families affect other actin cytoskeleton-dependent processes such as axonal growth and neuronal function [106, 109, 110]. Accordingly, consistent with increased MDV abundance and putative increase in sGTPase prenylation, DEP enrichment was observed in synaptic and sGTPAse signaling as well as vesicle-mediated transport, which is necessary for increased RPE-ChC cycling of LPPs.

Increased abundance of AP3B1 (adaptor-related protein complex 3, beta 1 subunit), which was only detected in APOB100 Tg mice, can also affect the RPE-ChC cycling of LPPs as AP3B1 is involved in intracellular vesicle transport between the Golgi and lysosomes/melanosomes [111]. In the eye, *Ap3b1* was shown to regulate the ocular melanosome biogenesis, including that in the RPE, with genetic *Ap3b1* abrogation leading to oculocutaneous albinism [112]. Notably, since energy is required for vesicle transport, there was a simultaneous increase in abundance of proteins involved in TCA cycle and respiratory electron transport (Fig. 11).

MID1IP1 (Mid1-interacting protein 1 or Mig12) is another protein that was only detected in APOB100 Tg mice (Fig. 11). MID1IP1 stabilizes microtubules and enhances fatty acid synthesis by stimulating the polymerization and activity of acetyl-CoA carboxylase (ACC), catalyzing the first committed step in fatty acid biosynthesis [113, 114]. MID1IP1 mRNA is regulated by SREBP-1a (sterol regulatory element-binding protein 1a), a potent activator of all SREBP-responsive genes, including cholesterologenic (e.g., MVD) and those involved in fatty acid synthesis [115]. Accordingly, consistent with increased MID1IP1 abundance and putative SREBP regulation, DEP enrichment was observed in the metabolism of different lipids, including fatty acids (Fig. 11). Thus, retinal proteomics provided some mechanistic links that need to be further investigated.

In addition, apolipoprotein O (APOO) was found for the first time in the retinal proteome of APOB100 Tg and WT mice (Table 2). APOO was initially detected in the hearts of diabetic dog as a novel apolipoprotein conserved among different species—mice, rats, pigs, dogs, and humans [116]. APOO is expressed in many tissues and is secreted as a 55 kDa chondroitin-containing proteoglycan. APOO secretion was found to depend on the MTTP (microsomal triglyceride transfer protein) activity [116], suggesting that APOO and APOB are probably secreted through the same pathway. In the systemic circulation, APOO is mainly present in the non-lipoprotein fraction as well as HDL and to a lesser extent in LDL and VLDL. APOO was suggested to promote cellular cholesterol efflux as efficiently as APOA1 and protect the diabetic human heart from excessive lipid storage [116]. Since the APOO expression in the retina has never been reported before, its retinal significance remains to be established.

In summary, studies of APOB100 Tg mice demonstrate that increases in the serum LDL content and the LDL/HDL ratio do not necessarily lead to a change in retinal cholesterol uptake from the systemic circulation and retinal in situ biosynthesis. This is likely due to several compensatory responses: an additional higher density HDL subpopulation and increased serum albumin content associated with the lower density HDL subpopulation; increased LPP cycling between the RPE and ChC; and only limited cholesterol traffic on LPPs from the RPE to the neural retina. The consequence of these responses and perhaps other, still need to be identified processes, is lipid deposition, but not yet drusen or basal linear deposits, in the RPE-BrM region of APOB100 Tg mice and their impaired overall retinal function.

## Data Availability

All the original data related to the figures in this article are available without undue reservation upon reasonable request to the corresponding author.

## Abbreviations

AMD: Age-related macular degeneration
au: Arbitrary units
BrM: Bruch’s membrane
ChC: Choroidal circulation
D_2_O: Deuterated water
D_7_-cholesterol: Deuterated cholesterol
DEP: Differentially expressed proteins
EC: Esterified cholesterol
ERG: Electroretinography responses
FA: Fluorescein angiography
FCED: Fat- and cholesterol-enriched diet
ICG: Indocyanine green
ICGA: Indocyanine green angiography
LFQ: Label-free quantification
LPP: Lipoprotein particle
PBS: Phosphate buffered saline
RPE: Retinal pigment epithelium
sGTPases: Small GTPases
SD-OCT: Spectral domain optical coherence tomography
TC: Total cholesterol
TEM: Transmission electron microscopy
UC: Unesterified cholesterol
WT: Wild type
